# Influence of Polymer Characteristics on the Self-Assembly of Polymer-Grafted Metal–Organic Framework Particles

**DOI:** 10.1021/acsnano.2c05175

**Published:** 2022-10-17

**Authors:** Kyle Barcus, Po-An Lin, Yilong Zhou, Gaurav Arya, Seth M. Cohen

**Affiliations:** † Department of Chemistry and Biochemistry, University of California, San Diego, La Jolla, California92093, United States; ‡ Department of Mechanical Engineering and Materials Science, Duke University, Durham, North Carolina27710, United States

**Keywords:** polymer brushes, polymers, metal−organic frameworks, self-assembly, molecular dynamics simulations, coarse-grained model

## Abstract

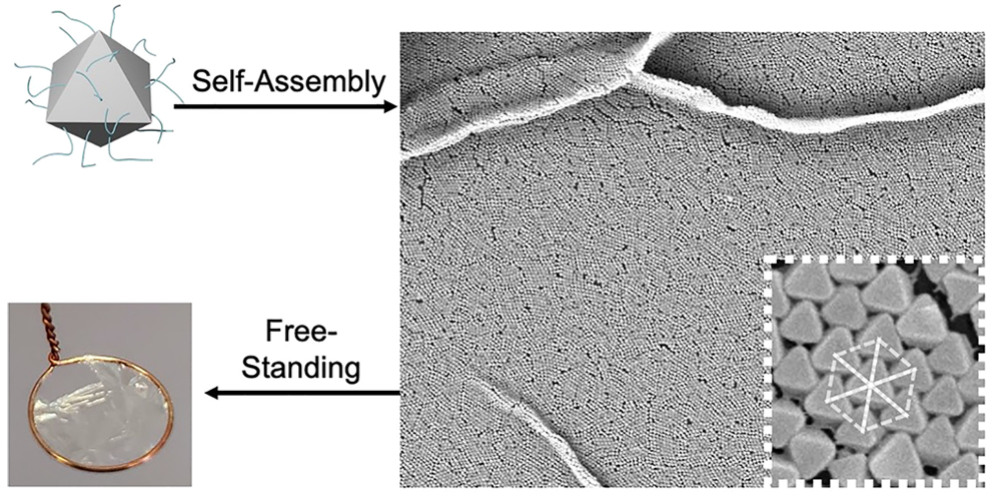

Polymer-grafted metal–organic frameworks (MOFs) can combine the properties of MOFs and polymers into a single, matrix-free composite material. Herein, we examine polymer-grafted MOF particles (using UiO-66 as a model system) to examine how the molecular weight, grafting density, and chemical functionality of the polymer graft affects the preparation of free-standing self-assembled MOF monolayers (SAMMs). The physical properties of the monolayers are influenced by the choice of polymer, and robust, flexible monolayers were achieved more readily with poly(methyl acrylate) when compared to poly(methyl methacrylate) or poly(benzyl methacrylate). Molecular dynamics simulations were carried out to provide insights into the orientation and ordering of MOFs in the monolayers with respect to MOF size, graft length, and hydrophobicity. The relationship between molecular weight and graft density of the polymer brush was investigated and related to polymer brush conformation, offering design rules for further optimizations to balance mechanical strength, MOF weight fraction, and processability for this class of hybrid materials.

## Introduction

The development of polymer composites with metal–organic frameworks (MOFs) offers a way to combine the properties of MOFs with the physical processability of polymers.^1−4^ However, the frequent incompatibility between the MOF particles and the polymer matrix may cause the particles to aggregate or produce brittle, fragile materials that lack the synergy of desired properties.^3,5−7^ Improving the interactions between the MOF and polymer without compromising the MOF structure or accessible porosity remains a challenge.

One of the more promising methods of making MOF–polymer composites is postsynthetic polymerization (PSP), which copolymerizes organic monomers with functionalized MOF particles to create a composite material with covalent connectivity between the two components.^8^ This covalent polymerization approach usually relies on the presence of functional groups appended to the MOF ligands that are suitable to react with organic monomers.^9−11^ While PSP is a useful method, not all MOFs are compatible with the requisite functionalized ligands or the reaction conditions required for further modification. An alternative approach is the use of surface anchoring groups to adhere polymerizable motifs to the MOF crystallite surface. Yang et al. have shown surface immobilization to be an effective way to form stable interactions between MOFs and various small molecules and polymers.^12^

Previous work that described grafting-from approaches for growing polymer chains from MOF surfaces employed a catechol-appended chain-transfer agent (CTA) conjugate designed to adhere the CTA to the surface of MOF nanoparticles (presumably by metal–ligand interactions).^13^ This CTA acted as both an initiation site and a control agent for the growth of polymer chains using reversible addition–fragmentation chain transfer (RAFT) polymerization. Well-defined grafts of poly(methyl methacrylate) (pMMA) were grown from the MOF surface while maintaining high surface area and crystallinity of the underlying MOF particles. The MOFs used in these prior experiments included the Zr(IV)-based UiO-66 and UiO-66-NH_2_ (UiO = University of Oslo), as well as the Fe(II)-based MIL-88B (MIL = Materials Institute Lavoiser).^13^ The polymer-grafted UiO-66 nanoparticles were further self-assembled at an air–water interface to form self-assembled MOF monolayers (SAMMs). A distinct dependence of the SAMMs on polymer molecular weight was observed, with increasing molecular weight resulting in tighter packing and fewer membrane defects. At above 138 kg/mol, it was possible to obtain free-standing, unsupported SAMMs comprised only of hybrid UiO-66-polymer particles. However, the same procedure performed on different MOFs gave varying results. For example, the chemically and morphologically similar UiO-66-NH_2_ was incapable of forming free-standing monolayers, while rod-shaped, Fe(II)-based MIL-88B-NH_2_ made SAMMs similar to UiO-66. The disparate outcomes from these MOF–polymer composites suggested that polymer chain length is one, but not the only, factor influencing SAMM formation.

To this end, an in-depth investigation of MOF self-assembly is reported here, where variations in the MOF size, as well as the chemical nature and molecular weight of the polymer grafts were explored. For each size of MOF particle, three different vinyl polymers—poly(methyl methacrylate) (pMMA), poly(benzyl methacrylate) (pBnMA), and poly(methyl acrylate) (pMA)—were polymerized from the surface at different molecular weights. Each MOF–polymer combination was then self-assembled at an air–water interface to more thoroughly understand the multi-scale interactions that contribute to the success and failure of achieving free-standing SAMMs and the factors that govern the properties of these materials in general. Concurrently, molecular dynamics (MD) simulations using a coarse-grained (CG) model were used for elucidating the roles of polymer grafting and particle size in dictating the orientation and translational order of MOF particles in the assembled SAMMs.

## Results and Discussion

### Synthesis of UiO-66

**Figure 1:**
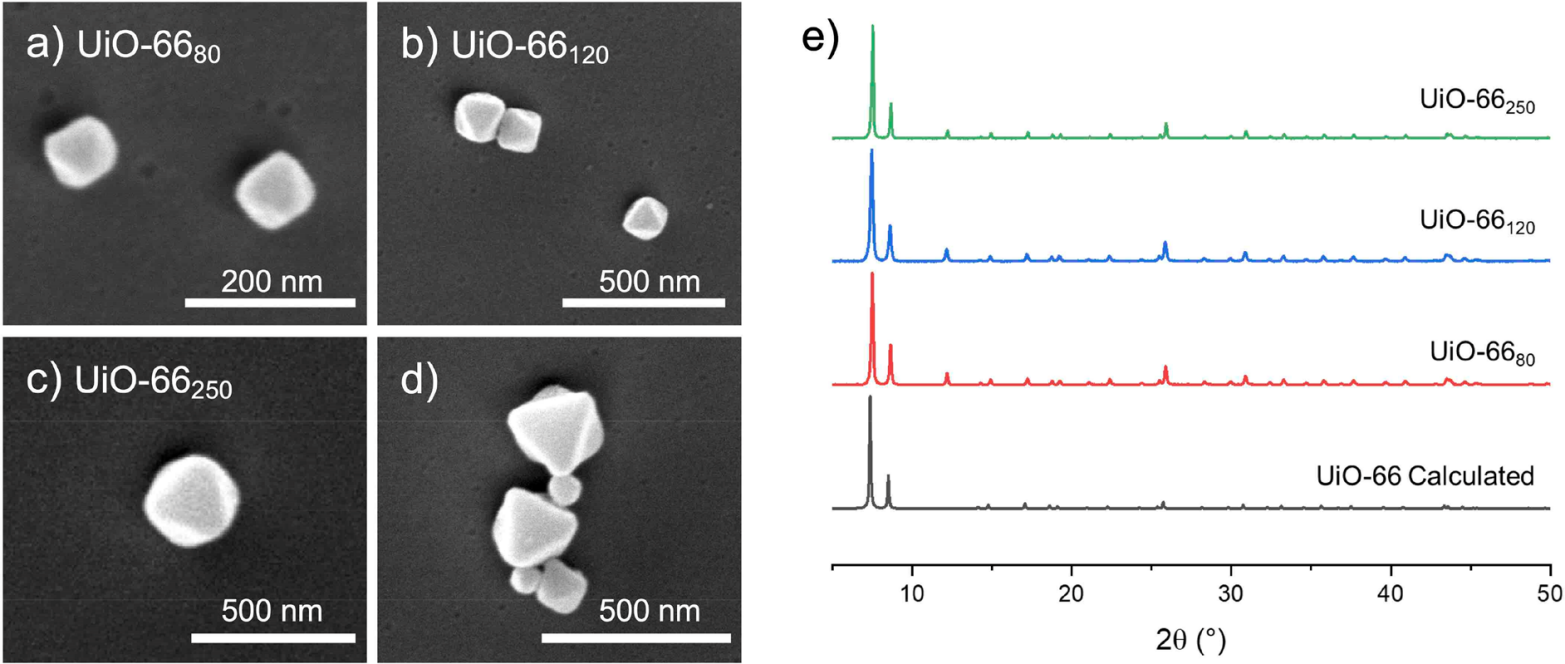
SEM images and PXRD of synthesized MOFs: (a) UiO-66_80_, (b) UiO-66_120_, (c) UiO-66_250_, and (d) mixture of all three MOFs displaying relative particle size (scale bars are 200 or 500 nm, as indicated). (e) Calculated and experimental PXRD patterns for MOF particles.

The synthesis of MOFs on a large scale while simultaneously controlling size, polydispersity, and morphology remains a significant challenge. For this study, a large-scale continuous-feed method introduced by Wang et al.^14^ was used, which afforded multi-gram quantities of UiO-66 in three distinct size regimes, termed UiO-66_*x*_ (*x* = the particle edge length in nm measured by scanning-electron microscopy, SEM) (Figure 1, Figure S1). Powder X-ray diffraction (PXRD) of the three sizes showed that all the MOFs exhibited good crystallinity (Figure 1). Nitrogen adsorption isotherms of the MOF particles were in good agreement with literature reports (Figure S2).^15^

### Surface Functionalization of MOFs with CTA

Control over the polymerization of different monomers is dependent on the structure of the CTA.^16^ To ensure control over a wide variety of monomers, two CTAs, 2-(dodecylthiocarbonothioylthio)-2-methylpropionic acid (DDMAT) and 4-cyano-4-[(dodecylsulfanylthiocarbonyl)sulfanyl]pentanoic acid (CDSPA), were used to control the polymerization of acrylates and methacrylates, respectively. A catechol group was introduced via the reaction of dopamine with the activated ester form of the CTA (Schemes S1 and S2). Surface functionalization of the MOF particles was performed using a biphasic mixture of an aqueous suspension of MOF particles in 10 mL water (20 mg/mL) and the CTA in 5 mL of chloroform (1 mg/mL) as previously reported (Scheme S3).^13^ Briefly, the aqueous and organic solutions were combined in a 50 mL centrifuge tube and vigorously mixed for 5 min using a vortex mixer to ensure adequate interfacial contact between the two solutions. The emulsion was broken with ethanol and the particles were collected by centrifugation. The solids were resuspended in ethanol and solvent exchanged by repeated centrifugation/dispersion cycles in ethanol, then solvent exchanged into DMSO to a final concentration of 80 mg/mL for further polymerization. After functionalization the MOF particles possess an orange/yellow color indicative of the presence of the CTA agent (Figure S3). Determination of the amount of CTA present on the surface of the MOF particles was attempted with several methods typically used in the literature for inorganic nanoparticles (e.g., ^1^H NMR, TGA, UV–vis). However, these methods proved ineffective for quantifying the CTA coverage due to the large excess of the terephthalic acid ligand (H_2_bdc) originating from the MOF that complicated these analyses. The UV–vis absorbance of the CTA at 300 nm overlapped completely with the absorbance from H_2_bdc, and TGA did not show a distinct mass loss between unfunctionalized and functionalized MOF particles. While ^1^H NMR of digested MOF particles could resolve the presence of the long alkyl chain of the CTA, the exact quantity of CTA present could not be determined with confidence.

### Surface-Initiated RAFT Polymerization of MMA from UiO-66

**Scheme 1:**
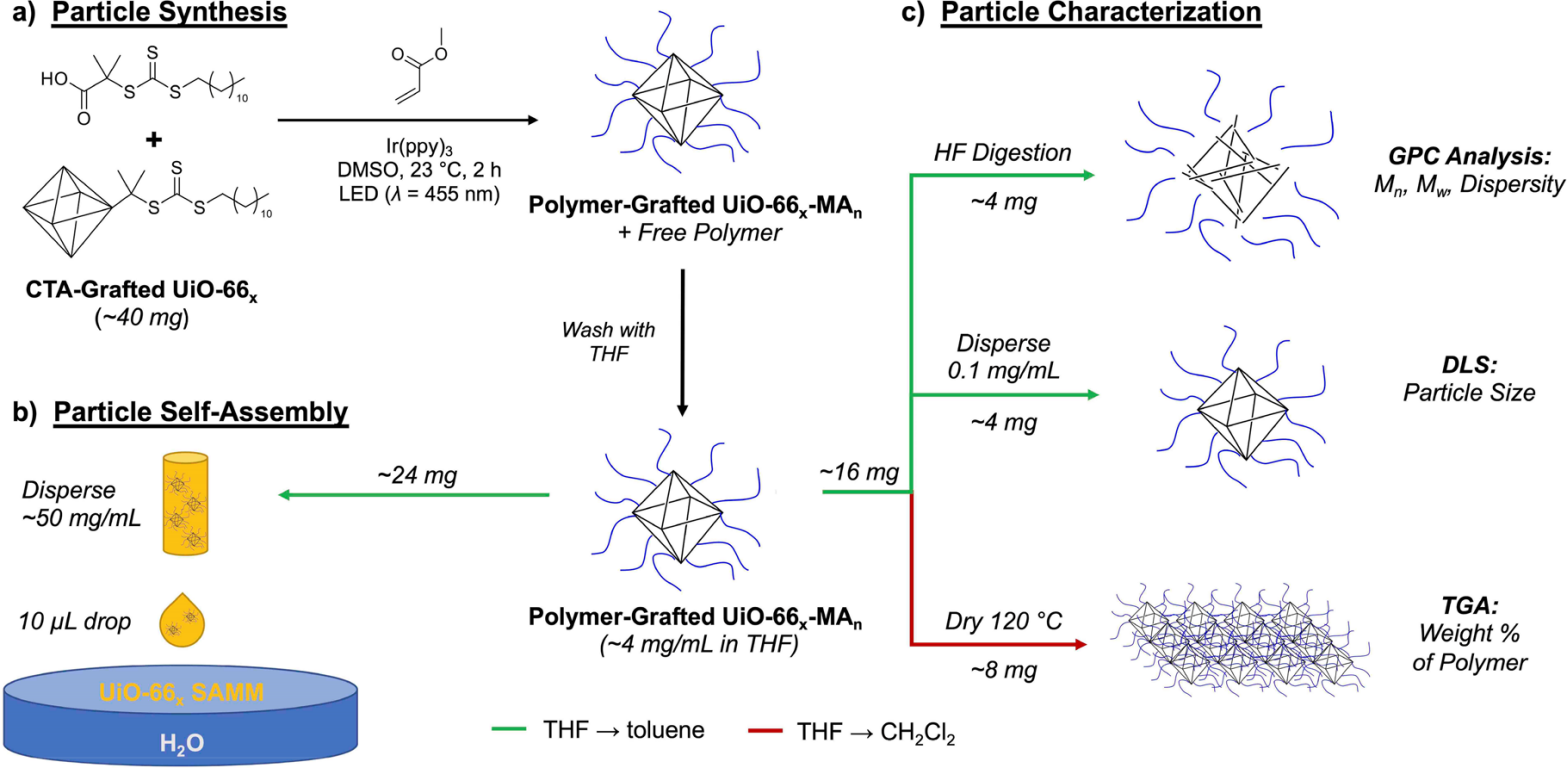
Workflow for Synthesis and Characterization of Polymer-Grafted MOF Particles and the Resulting Self-Assembled MOF Monolayers (SAMMs) Using UiO-66_*x*_-MA_*n*_ as an Example (a) Synthesis of polymer-grafted MOF particles via surface-initiated photoinduced electron-transfer reversible addition–fragmentation chain-transfer polymerization (SI-PET-RAFT). (b) Self-assembly of particle monolayers at an air–water interface. (c) Characterization of particles via GPC, DLS, and TGA. Green and red arrows indicate solvent changes from THF to toluene and CH_2_Cl_2_, respectively.

To analyze the effect of the polymer backbone and side-chain effects on self-assembly, we chose poly(methyl methacrylate) (pMMA), poly(benzyl methacrylate) (pBnMA), and poly(methyl acrylate) (pMA) as polymers with different physical properties. The polymer graft was synthesized by surface-initiated photoinduced electron transfer reversible addition–fragmentation chain transfer polymerization (SI-PET-RAFT) using Ir(ppy)_3_ as the photocatalyst under blue LED lights (Scheme 1, Figure S4). Free, unbound CTA without the anchoring catechol group was included in each polymerization to ensure efficient chain transfer and control from the surface.^17^ While the amount of CTA bound to the surface was unknown, the excess of free CTA was used to ensure that the polymerization on the surface is controlled regardless of the amount of surface CTA present. For acrylates, the same DDMAT CTA was used on both the surface (cat-DDMAT) and in solution (Scheme S4). However, better control was achieved in the polymerization of methacrylates when 4-cyano-4-(phenylcarbonothioylthio)pentanoic acid (CPADB) was used as the free CTA in solution instead of CDSPA due to the higher transfer constant of the former (Schemes S5 and S6).^16^ To check the effect of the MOF on the polymerization itself, a control experiment using methyl acrylate as the monomer was performed without MOF, with unfunctionalized UiO-66_120_, and DDMAT functionalized UiO-66_120_. Analysis of the free polymer shows that the presence of UiO-66 does not have a large effect on the polymerization (Table S2).

Each MOF/polymer combination was polymerized to different molecular weights to see how the length of the polymer chain relative to particle size affected the particle self-assembly and physical properties of the monolayer (Table S1). After polymerization, the particles were separated from ungrafted (e.g., free in solution) polymer via five cycles of centrifugation, decanting, and redispersion in THF. A small sample of the particles was removed for characterization while the remaining particles were solvent exchanged into toluene for self-assembly at an air–water interface (Scheme 1). The molecular weight of the surface-bound polymer was characterized by digesting the polymer-grafted MOF in HF/toluene followed by gel-permeation chromatography (GPC), while thermogravimetric analysis (TGA) was used to calculate the weight percent of the polymer relative to MOF.

**Figure 2:**
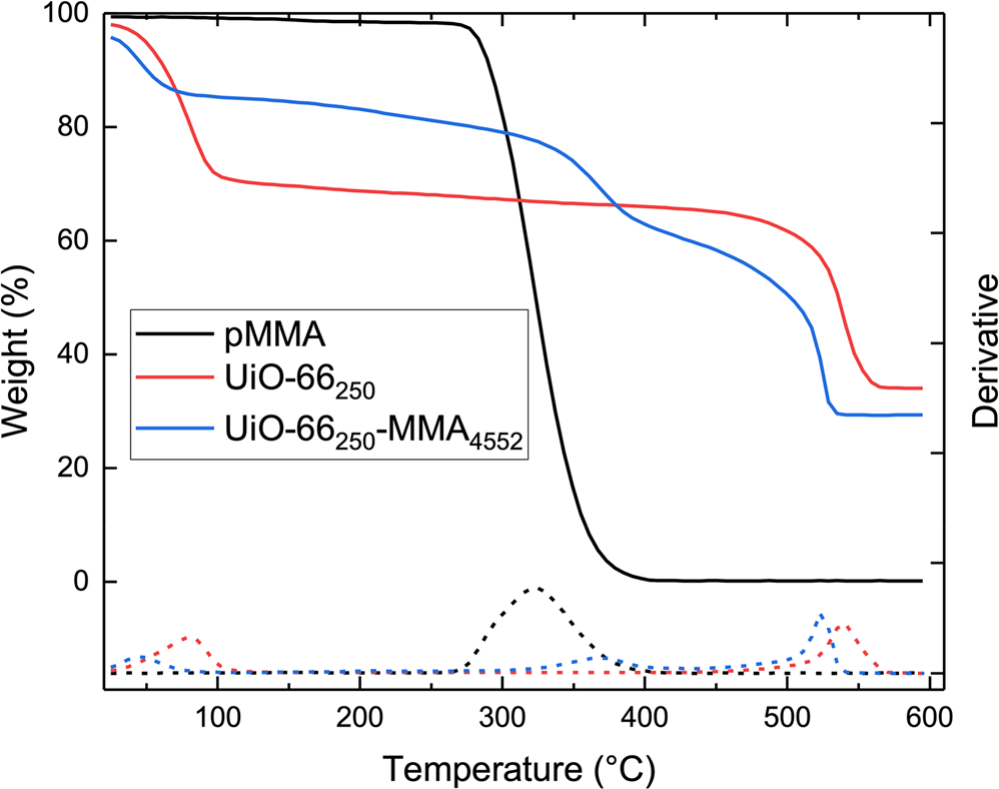
Representative TGA plot of weight percent (solid lines, left *y*-axis) and derivative of weight percent (dashed lines, right *y*-axis) for pMMA (black), UiO-66_250_ (red), and UiO-66_250_-MMA_4552_ (blue).

The porous and organic–inorganic nature of the MOF adds some complexity to the TGA analysis, and three distinct regimes of mass loss occur from 0 to 600 °C (Figure 2). The initial mass loss is due to the evaporation of solvent from the MOF pores, while the mass losses from 280 to 420 °C and 420 to 600 °C correspond to the degradation of the polymer and MOF, respectively. The remaining mass is residual ZrO, and by comparing these values, the amount of polymer relative to MOF can be determined.

**Figure 3:**
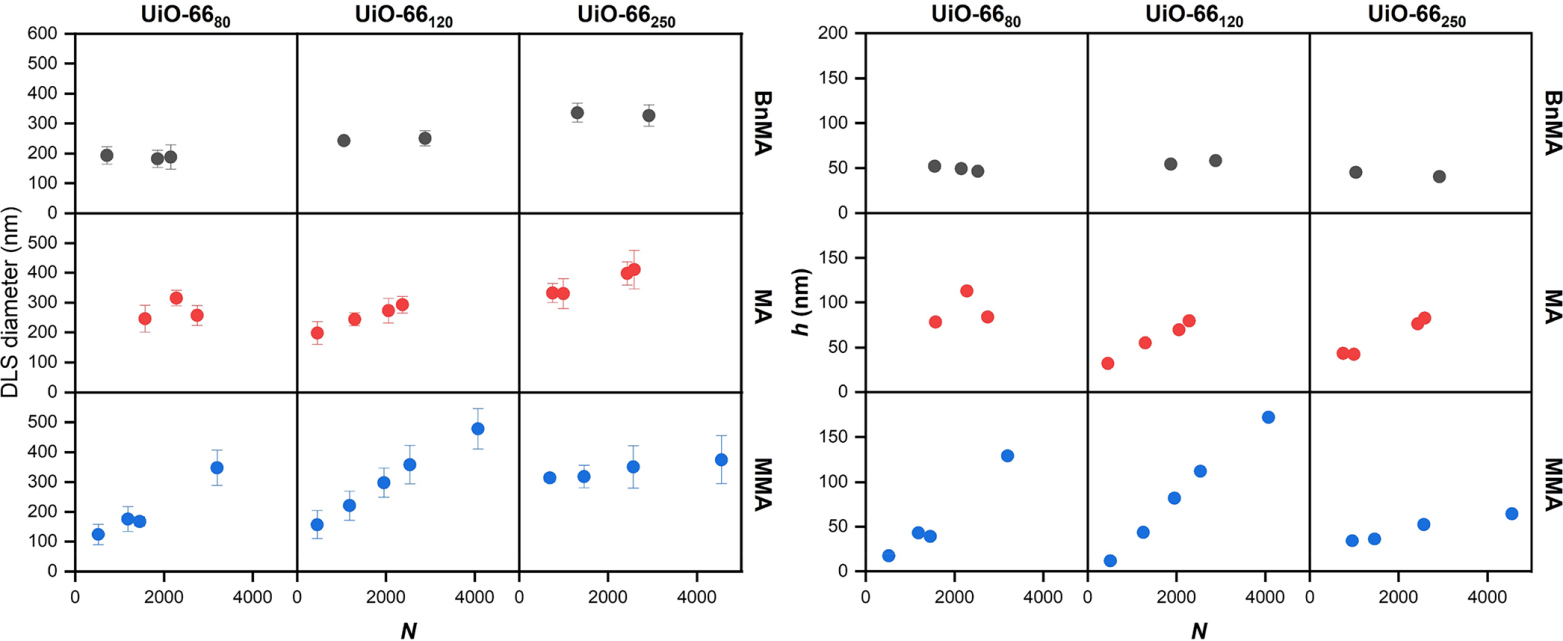
Left: Particle diameter as characterized by dynamic light scattering (DLS) in toluene with respect to the surface polymer length as determined by gel permeation chromatography (GPC). Error bars are the standard deviation of three independent measurements. Right: Relationship of the polymer brush height (*h*) determined by DLS as a function of increasing polymer length (*N*). Columns designate the size of the MOF particle and rows designate the monomer used. Each point is the average of three independent DLS measurements.

With the molecular weight and relative mass of the polymer obtained by GPC and TGA, respectively, the grafting density on the surface of the MOF can be estimated by the following equation:
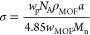
where *w*_p_ is the weight fraction of the polymer and *w*_MOF_ is the weight fraction of MOF as determined by TGA, *N*_A_ is Avogadro’s number, ρ_MOF_ is the density of UiO-66, *a* is the edge length of the octahedron, and *M*_n_ is the molecular weight of the surface-grafted polymer.^18^ The grafting densities of all samples are shown in Table S1. The brush height of the polymer grafts was determined by subtracting the radius of the core particle, *r*_0_, from the radius of the polymer-grafted MOF nanoparticle (PGMN) obtained by dynamic light scattering (DLS) of the particles in toluene. It should be noted that the size measured by DLS (Figure 3) is representative of a sphere with equivalent Brownian motion, which does not account for the octahedral shape of the MOF particles.^19^ To simplify the calculations, a sphere of intermediate radius to the MOF particle was assumed as the core radius and subtracted from the radius determined by DLS to get the brush height, *h* (see Supporting Information for a detailed explanation, including Figure S5).

The brush height, *h*, as a function of degree of polymerization, *N*, shows different scaling depending on the size of the MOF and the grafted polymer (Figure 3). In the case of UiO-66_*x*_-BnMA_*n*_, the samples show no change in brush height at any values of *N*, whereas UiO-66_*x*_-MMA_*n*_ and UiO-66_*x*_-MA_*n*_ show a linear increase in all cases excluding UiO-66_80_-MA_*n*_. These results are shown primarily to illustrate the potential of these combined methods to analyze the polymer graft on a MOF surface beyond simple molecular weight characterization, as clear variations can occur between samples with similar *N*. However, the current data set is not sufficient to develop a robust physical model of the polymer microstructure across all the different variables, and more data is needed to comprehensively understand how these MOF–polymer brush materials compare to other polymer grafted nanoparticle systems.

### Self-Assembly at the Air–Water Interface

The self-assembly of the polymer-grafted MOF particles into SAMMs was investigated. The self-assembly at the air–water interface was performed by adding a 10 μL drop of a 10 wt% suspension of the polymer-grafted MOF particles in toluene to a layer of water in a plastic Petri dish (diameter = 55 mm). The drop immediately spread to the edge of the dish and was quickly covered with a lid to prevent disturbance from the evaporation process and air turbulence. After 10 min the lid was removed, revealing an iridescent film. The monolayer was then suspended on a 7 mm loop of copper wire, which held a drop of water supporting the monolayer (Scheme S7). The wire loop was then suspended to air-dry, after which the film either broke or remained suspended as a free-standing monolayer.

As previously reported, polymer-grafted MOFs with low molecular weight (*N* < 1000) gave monolayers with poor mechanical properties and easily fractured when disturbed. Depending on the polymer used, as MW was increased further the monolayers began to behave more like polymeric films, with large areas of the film responding to localized stress indicating a significant level of entanglement between the particles. Films formed from pBnMA or pMMA were brittle and easily fractured when disturbed. Of these two polymers, only pMMA was able to form free-standing membranes at higher molecular weights (*N* > 4000). However, when the polymer was changed to pMA, a significant difference in membrane forming behavior was observed. For *N* > 1000 with pMA, the monolayer films were extremely tough and flexible, exhibiting none of the brittleness of pMMA. The monolayers formed from polymer-grafted MOFs with pMA were strong enough that removal of sections of the films for SEM imaging was nearly impossible, with the entire film delaminating from the water surface (Video S1).

**Figure 4:**
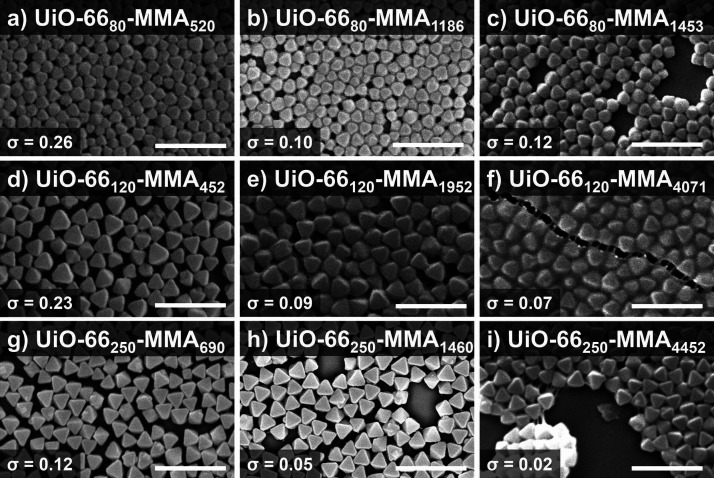
SEM images of SAMMs with pMMA. (a–c) UiO-66_80_-MMA_*n*_ (scale bars 500 nm). (d–f) UiO-66_120_-MMA_*n*_ (scale bars 500 nm). (g, h) UiO-66_250_-MMA_*n*_ (scale bars, 1 μm). Grafting density values (σ) are shown.

To understand the origin of these pronounced differences in film properties with varying polymer type and length, sections of each monolayer were transferred from the water surface to a glass coverslip for SEM imaging (Figure 4, Figures S6–S8). In the case of UiO-66_80_-MMA_*n*_, the presence of polymer coating on the exterior of the particles is visible for UiO-66_80_-MMA_1453_, but the loose packing indicates that the chains fail to entangle enough to prevent separation (Figure 4c, Figure S6). At a comparable molecular weight, UiO-66_250_-MMA_1460_ shows little visible polymer present on the surface (Figure 4h). At *N* > 4000, densely packed films form for UiO-66_120_ and the polymer brushes are clearly entangled enough between particles to show distinct crazing as cracks form through the material (Figure 4f,i). However, only UiO-66_120_-MMA_4071_ remained a free-standing membrane while the UiO-66_250_-MMA_4452_ monolayer fractured. As both particles have similar polymer lengths and grafting densities, this effect is presumed to be a result of the significantly larger particle size for UiO-66_250_-MMA_4452_. The larger particle size results in larger gaps between MOFs that the polymer chains must bridge to hold the particles together, and the chains are not able to form substantial entanglements across these interstitial spaces for UiO-66_250_-MMA_4452_. We also observed that as the MOFs become smaller or are grafted with longer chains, the particles lose some of their translational and orientational order in the films (for instance, compare Figure 4d with 4f). As the MOF particles become smaller, it is more challenging to obtain uniformly sized particles (compare Figure 4a with 4g), which could also contribute to some loss in order. Even if the absolute variations were the same across all particle sizes, the small particles will exhibit larger *relative* variations in size (the primary factor dictating their ordering) compared to large particles. We also noted some rounding off at the vertices of the MOF particles. Because the relative effect of curvature is stronger on smaller particles than on larger ones (even if the absolute curvature was the same), this effect could also contribute to the increasing disorder with decreasing particle size.

**Figure 5:**
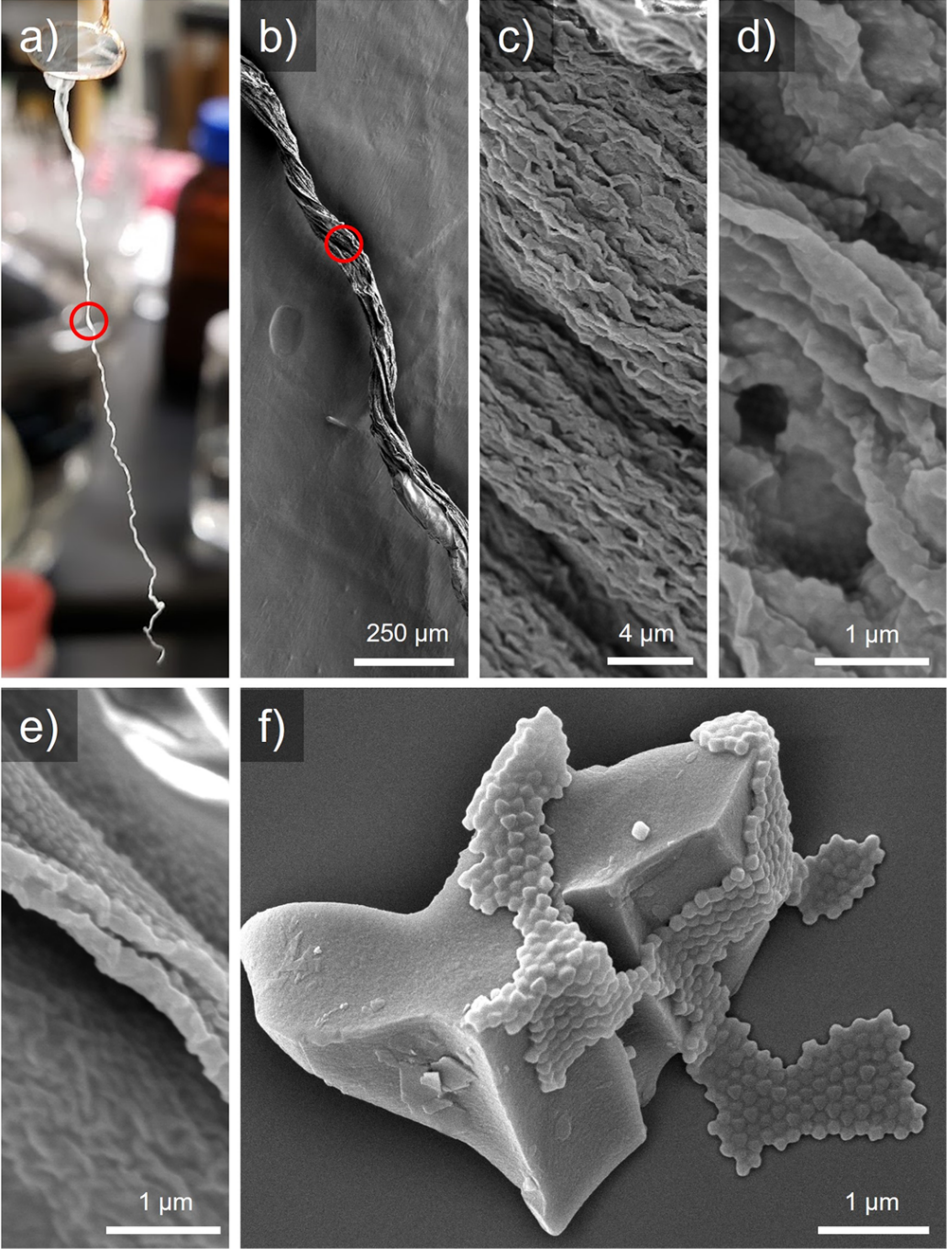
(a) Delaminated monolayer of UiO-66_120_-MA_1294_ in dry fiber form. (b–e) SEM images of fiber at increasing magnification. (f) SEM of monolayer on small glass fragment (irregular shaped solid particle) showing ability to conform to various surface curvatures while retaining ordered monolayer.

Compared to UiO-66_*x*_-MMA_*n*_, images of the highly ductile UiO-66_*x*_-MA_*n*_ monolayers showed significant polymer entanglement at much lower molecular weights (Figure S7). Free-standing films were achieved for all monolayers with *N* > 1000 regardless of particle size, indicating the mechanism for this improved mechanical strength is not a result of simply increasing molecular weight to a higher value. The grafting density and brush height are both higher at comparable molecular weights to the MMA grafts. It is not obvious what leads to the higher initial graft density in the MA polymerization as the graft density of initiator should be the same. One possible explanation is the acrylate polymerization in this particular system provides better control than methyl methacrylate.^20^ This would lead to more uniform growth at the initial stages of polymerization forming a dense brush at low molecular weights until steric crowding begins to prevent activation–deactivation by the CTA. This higher grafting density forces the polymer chains to extend further from the surface. As molecular weight increases, the increased grafting density results in more entanglements per particle, which prevents the SAMM from cracking during the drying process resulting in a highly interconnected, flexible film. SEM images of the delaminated film in Video S1 shows that the fiber formed is comprised from a single monolayer twisted and folded into itself (Figure 5a–e).

This macroscopic flexibility extends to the microscale as well, with SEM images of the monolayer on a small glass fragment showing the film can tightly adhere to both convex and concave surfaces of high curvature without breaking the ordered monolayer structure (Figure 5f). These results are encouraging when considering future applications, as the films can be applied to a wide variety of substrates with rough surface features without compromising the monolayer.

As a representative example of the polymer-coated MOF particles, the accessible surface area of UiO-66_250_-MMA_2566_ was measured using N_2_ gas. The BET surface area of UiO-66_250_-MMA_2566_ was determined to be 885 m^2^/g; by comparison, the unmodified UiO-66_250_ material gave a surface area of 1442 m^2^/g (Figure S9). TGA shows UiO-66_250_-MMA_2566_ is 20% polymer by mass (Table S1); therefore, the expected surface area of UiO-66_250_-MMA_2566_ based on the weight percent of the MOF and the surface area of the unmodified particles (80% of 1442 m^2^/g) is ∼1150 m^2^/g. This data suggest that UiO-66_250_-MMA_2566_ retains ∼75% of the expected surface area.

### Simulations of MOF Orientation and Assembly

**Figure 6:**
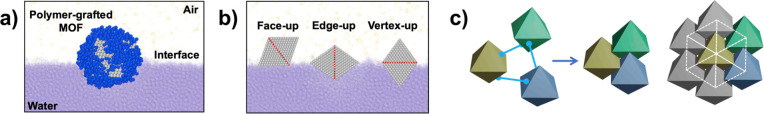
Modeling and simulation of MOF orientation and assembly at an interface. (a) Simulation setup showing the coarse-grained model of polymer-grafted MOF trapped at an air–water interface. (b) Side view of the three idealized orientations of MOFs shown without the grafts. Red dotted lines represent the square base of a regular octahedron. (c) Schematic illustrating the formation of face-to-face contacts between MOFs leading to hexagonal packing (highlighted by white dotted lines).

To understand the observed changes in the orientational and translational order of MOFs with respect to their size, graft type, and MW, coarse-grained molecular dynamics (CG MD) simulations of the MOF–air–water system were performed (Figure 6). Analogous to the experiments, the effects of varying MOF edge length *L*_MOF_, graft length *L*_g_, and graft hydrophobicity λ were examined. As detailed below, the parameter λ describes the relative strength of polymer–solvent to solvent–solvent interactions, where λ = 0 indicates strongly hydrophobic chains and λ = 1 indicates strongly hydrophilic chains.

**Figure 7:**
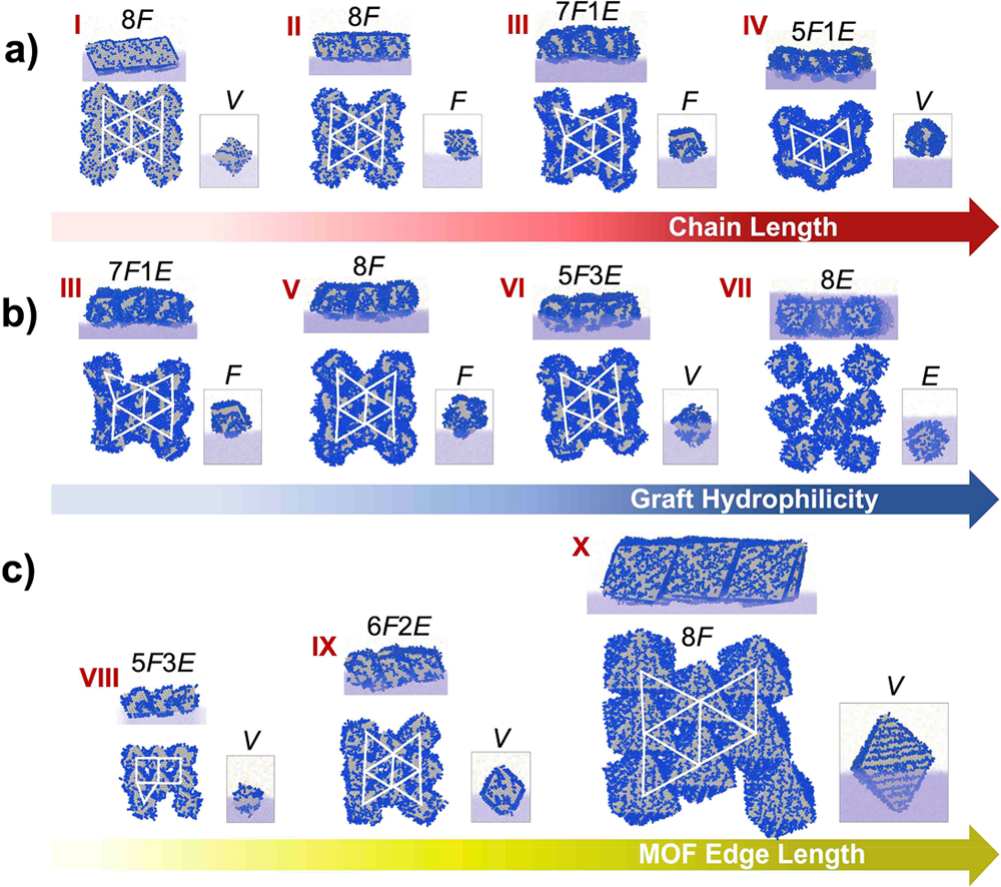
MOF orientation and assembly predicted by simulations. Side and top views of structures assembled with MOFs of increasing (a) graft lengths *L*_*g*_ = 1σ_CG_, 2σ_CG_, 4σ_CG_, and 6σ_CG_ for fixed MOF size *L*_MOF_ = 13σ_CG_ and graft hydrophilicity λ = 0.2, where σ_CG_ is the basic length scale in our coarse-grained model; (b) hydrophilicity of graft monomers λ = 0.2,0.4,0.6, and 1 for fixed MOF size *L*_MOF_ = 13σ_CG_ and graft length *L*_g_ = 4; and (c) edge length *L*_MOF_ = 9σ_CG_, 13σ_CG_, and 28σ_CG_ for fixed *L*_g_ = 2σ_CG_, λ = 0.4. Insets show the orientation adopted by a single, isolated MOF particle (E = “edge-up”; F = “face-up”; V = “vertex-up”). The orientations adopted by each MOF particle in the assembled structure were enumerated and indicated as *x*F*y*E (*x* and *y* are integers), shown immediately above each structure. White triangles highlight the hexagonal packing of particles.

First, the orientation of *individual* MOFs at the interface (Figure 7, insets) were examined, which were classified as “face-up”, “edge-up”, or “vertex-up” based on the interface-projected areas of the MOFs (Figure 6b, Figure S10). Based on energetic arguments, octahedral particles that interact similarly with fluids on both sides of the interface should reside symmetrically about the interfacial plane and adopt a vertex-up orientation, which maximizes the occluded area of the energetically unfavorable interface.^21,22^ Indeed, we find that MOFs with moderately hydrophilic grafts stay close to the interfacial plane and exhibit vertex-up orientation (labeled VI, Figure 7). Similarly, MOFs with short hydrophobic grafts, where the hydrophilic surface of the MOF balances out the hydrophobicity of the grafts, also reside close to the interface and exhibit vertex-up orientation (I, VIII–X, Figure 7). However, as the grafts become more hydrophobic and long enough to screen out favorable MOF–water interactions (II, III, V, Figure 7), the MOFs shift from the water to the air phase and exhibit face-up orientation, thereby maximizing the occluded area of the interface while minimizing the unfavorable graft–water interactions. Interestingly, when the grafts become very long, the MOFs almost completely detach from the water phase and begin to exhibit vertex-up orientation (IV, Figure 7). This configuration best avoids contact between the grafts and the water phase, as the grafts are generally depleted at the MOF tips. On the other hand, strongly hydrophilic grafts cause the particles to fully submerge into the water phase and adopt the edge-up orientation (VII, Figure 7), which allows some area of the interface to be occluded while maximizing favorable graft–water interactions.

Next, the assembly of *multiple* MOF particles was examined at the interface. Our simulations revealed that the MOFs assembled primarily via face–face contacts (Figure 7), leading to the hexagonal packing observed in the experiments. Interestingly, many of the assembled structures were composed of face-up oriented MOFs, even when they preferred to be vertex-up or edge-up in isolation (I, IV, VI, VIII–X, Figure 7). This can be explained by the large free energy gained from face-to-face contacts enabled by the face-up orientation of the MOFs, which compensates for the loss in free energy due to reorientation (Figure 6c); if the MOFs had remained vertex-up, the free energy gained from tip–tip contacts would be small due to the small area of interactions.^22^ This finding is also consistent with our previous study on polymer-grafted nanocubes.^23^ With this ability to mediate face-to-face contacts, a single MOF can mediate interactions (via its six lateral facets) with six adjacent MOFs, leading to the observed hexagonal packing arrangement of the MOFs. Importantly, these results also suggest that interparticle interactions must be very strong in these MOF systems, prevailing over interfacial interactions that would otherwise have led to assemblies with different orientations.

The degree of hexagonal ordering of MOFs and the homogeneity of their orientation in the self-assembled structures depended strongly on the graft length, their hydrophilicity, and particle size. In general, we found that as the grafts became longer, the interparticle distance increased, and the MOFs lost their octahedral character, leading to more disordered packing (Figure 7a). This finding is consistent with our experimental observations (for an example, see UiO-66_250_-MA_*n*_, Figure S11). Decreasing the hydrophobicity of the grafts also led to more disordered packing, and eventually no assembly at all for highly hydrophilic grafts, which were strongly wetted by the surrounding water molecules (Figure 7b). Lastly, increasing the MOF size led to more uniform orientations (face-up) and packing (hexagonal) of the MOFs (Figure 7c), also consistent with our experiments when using benzyl methacrylate (Figure S10). The assemblies with the smallest MOFs considered here (UiO-66_80_-BnMA_715_) exhibited large fluctuations in particle orientation and a more square-like rather than hexagonal order, whereas those with the largest MOFs (UiO-66_250_-BnMA_1309_) exhibited hexagonal ordering with uniformly face-up particles (Figure S12). Interestingly, we observed that the hexagonal order appeared before the orientational order as the size of the MOFs was increased. Overall, our simulations suggest that the polymer grafts need to be sufficiently short relative to the particle size and sufficiently hydrophobic to exhibit orientational and translational order, which is in contrast to those found to promote the assembly of robust SAMMs at the air–water interface.

## Conclusions

UiO-66 octahedral nanoparticles were prepared in three distinct size regimes and further functionalized with pMMA, pBnMA, and pMA via a grafting-from approach using a SI-PET-RAFT polymerization procedure. The effects of particle size, polymer type, and polymer length at an intermediate grafting density (σ ≈ 0.02–0.2 chains/nm) were explored with respect to the physical properties of the self-assembled monolayers. Increasing polymer length led to increased interparticle chain entanglements and significant improvements in the physical stability of the resulting monolayers, with diminishing improvements as particle size increases. Switching from pMMA to pMA, significantly altered the properties of the monolayers to reflect the bulk polymer, with glassy pMMA grafts giving more brittle monolayers and rubbery pMA grafts producing tough, flexible films. Free-standing monolayers were easier to achieve at an intermediate particle size (120 nm) and the ideal combination of factors for mechanically robust SAMMs was found using intermediate 120 nm particles grafted with high *M*_*n*_ pMA. Simulations provided additional insights into the orientation and ordering of MOFs within the films as a function of particle size, graft length, and hydrophobicity. These polymer-grafted, self-assembling MOF particles may find further application in ultrathin membranes for separations, protective coatings, and optical films.

## Methods

### Synthesis of UiO-66_*x*_

UiO-66 was prepared using a continuous addition method as previously reported.^14^ The synthesis of UiO-66_*x*_ (*x* = the particle edge length in nm measured by SEM) at 5 L scale was carried out at 120 °C under atmospheric pressure in DMF using formic acid as a modulator. Two separate 30 mM stock solutions were prepared in 5 L vessels. The terephthalic acid (H_2_bdc) solution was prepared with 22.5 g of H_2_bdc, 4.05 L of DMF, and 450 mL of formic acid, while the ZrOCl_2_·8H_2_O was prepared with 45 g of ZrOCl_2_·8H_2_O in 4.5 L of DMF. The reaction procedure is as follows (Figure S1). An initial 100 mL of the ZrOCl_2_·8H_2_O solution was added to a 5 L round-bottom flask at 120 °C, then both the ZrOCl_2_·8H_2_O (mM) and H_2_bdc stock solution were separately delivered with feed rate of 12 mL/min for 5 min. The feed rate was accelerated to 32 mL/min for 55 min. After this first addition, 2.5 L of the reaction solution was removed from the reactor to obtain the first product, UiO-66_80_, and then 1.5 L of metal stock solution and 1.5 L of ligand stock solution were further added into the remaining reaction solution at 30 mL/min for 50 min. Then 3 L of reaction solution was collected form the reactor to obtain the second product, UiO-66_120_. Finally, 1.55 L of metal stock solution and 1.55 L of ligand stock solution were added into the reactor within 1 h at 25.8 mL/min, and the remaining reaction solution (3.7 L) was collected as the third product UiO-66_250_. All products were first centrifuged (8000 rpm, 30–60 min) and washed with 2 × 40 mL of DMF, and then solvent exchange was performed by washing with 3 × 40 mL of MeOH. The MOFs were left suspended in MeOH at ∼20 mg/mL until further use. A fraction of this suspension was removed and dried to determine the exact weight percent of the suspended particles. For PXRD and N_2_ sorption experiments the samples were dried in vacuum at 120 °C for 24 h.

### Surface Functionalization of UiO-66_*x*_ with cat-CTA

A 50 mL centrifuge tube was prepared with 200 mg of UiO-66_*x*_ in MeOH, centrifuged (8000 rpm, 15 min) to collect the particles, and redispersed in 10 mL of water. A separate vial was prepared with 10 mg of either cat-CDSPA or cat-DDMAT dissolved in 5 mL of CHCl_3_ and added to the aqueous MOF suspension. The biphasic mixture was vortexed for 5 min, and then 20 mL of EtOH was added to form a homogeneous suspension. The particles were collected by centrifugation (8000 rpm, 15 min), washed via repeated dispersion/centrifugation cycles with EtOH (2 × 25 mL, 30 min immersion each), followed by DMSO (3 × 20 mL, 30 min immersion each), and finally suspended in DMSO at a concentration of 80 mg/mL.

### SI-PET-RAFT Polymerization of MA from UiO-66_*x*_

A 10 mL round-bottom flask was charged with a magnetic stir bar, 2.5 mL DMSO, and 500 μL of an 80 mg/mL stock solution of UiO-66_*x*_-DDMAT suspended in DMSO. The solution was constantly stirred while DDMAT (3.38 mg, 9.3 μmol, 1 equiv) and Ir(ppy)_3_ (12.1 μg, 0.018 μmol,.002 equiv) were added (from 10 and 1 mg/mL DMF stock solutions, respectively). Methyl acrylate (1.68 mL, 18.5 mmol, 2000 equiv) was then added dropwise, after which the suspension was left without stirring for 5 min to ensure that the MOF particles had not aggregated and settled. The reaction was then sealed tight with a rubber septum secured with a copper wire and degassed with Ar for 30 min before transferring to a home-built blue light LED photoreactor (Figure S4) and irradiated until stirring ceased. The reaction was diluted with 40 mL of THF, transferred to a 50 mL centrifuge tube, and the particles were collected by centrifugation. The particles were then washed with 5 × 40 mL of THF until analysis of the supernatant by gel permeation chromatography (GPC) showed no free polymer present. The particles were resuspended in 10 mL of toluene and transferred to a 15 mL centrifuge tube before dividing further into samples for characterization and self-assembly (Scheme S3). The surface-bound polymer was analyzed by removing 1 mL of the toluene suspension and digesting the material with 10 μL HF (48% H_2_O) and 500 μL DMSO for 1 h, then partitioning the solution between 7 mL H_2_O and 3 mL toluene. The polymer remaining in the toluene layer was isolated by transferring the toluene to a vial, evaporating the solvent under high vacuum at 30 °C, and dissolving the residue in 1 mL THF for analysis by GPC.

### Self-Assembly at the Air–Water Interface

The polymer-grafted particles were suspended at 10–20 wt % in toluene (∼500 μL). A 10 μL drop of the solution was gently dropped on the surface of water in a 55 mm diameter plastic Petri dish, which was quickly covered with a lid. After the toluene had fully evaporated (∼10 min), the monolayer was lifted onto the surface of a glass slide for imaging. To prepare free-standing monolayers, a copper loop was prepared by wrapping copper wire (diameter ∼0.7 mm) around a 1 mL plastic syringe barrel. The loop was removed and placed under the water surface, then quickly lifted from underneath the monolayer, suspending a drop of water with the film floating on the surface. The loop was hung to dry in air, leaving a thin film of the MOF–polymer membrane, which was then imaged by SEM.

### Coarse-Grained (CG) Model of the MOF Interface System

A CG model previously used for studying polymer-grafted nanoparticles at polymer interfaces was adapted for treating polymer-grafted MOFs at an air–water interface.^24,25^ Briefly, the MOFs were modeled as rigid octahedra constructed out of a lattice of CG beads of size σ_CG_. Octahedra of edge lengths 9σ_CG_, 13σ_CG_, and 28σ_CG_ were explored, corresponding to experimental MOFs of edge lengths 80, 120, and 250 nm. The polymer grafts were modeled as chains of CG beads (also of size σ_CG_) representing short segments of the polymer chain.^26^ Adjacent beads in the chain were connected via finitely extensible nonlinear elastic (FENE) springs and interact with each other via a short-ranged repulsive Weeks–Chandler–Anderson (WCA) excluded-volume potential.^27^ The grafts were attached uniformly to all facets of the MOF particles at a grafting density of 0.3 chains/σ_CG_^2^. To study the effects of the degree of polymerization of the grafts examined experimentally, chain lengths of 1, 2, 4, and 6 beads were investigated. The water and air phases were also treated using CG beads, which interact with each other *within* the same phase via an attractive Lennard-Jones (LJ) potential of size σ_CG_ and energy ε and *across* the phase with a repulsive WCA potential. The two fluids were maintained at densities of 0.4 and 0.02 beads/ σ_CG_^3^, which led to stable gas- and liquid-like phases and a sufficiently large surface tension between them at the simulated temperature. The remaining interactions between beads comprising the solvent, MOF particles, and polymer grafts were also treated using a combination of LJ and WCA potentials, depending on their mutual miscibility. For convenience, we considered the same size and energy parameters σ_CG_ and ε for these potentials, except for those describing the interactions between polymer graft beads in the water phase. These interactions were treated using an LJ potential with an adjustable energy parameter λε, where λ was varied between a value of 0 signifying strongly hydrophobic chains to a value of 1 signifying hydrophilic chains.

### Molecular Dynamics (MD) Simulations

The LAMMPS program was used for carrying out MD simulations of polymer-grafted MOFs at the air–water interface.^28^ All simulations were carried out in the canonical (NVT) ensemble at a temperature of 0.7 ε/*k*_B_, where *k*_B_ is the Boltzmann constant. A velocity-Verlet algorithm with a time step of 0.002 (*m*σ_CG_^2^/ε)^1/2^ (*m* = mass of each CG bead) and a Nosé–Hoover thermostat with a time constant of 1.0 (*m*σ_CG_^2^ /ε)^1/2^ were used for integrating the equations of motion and controlling temperature. Two impermeable LJ walls were used to confine the air and water particles in the *z*-direction normal to the interface, while periodic boundary conditions were applied in the *x* and *y* directions parallel to the interface. To minimize the effect of the walls on the interface, the air and water layers were chosen to be sufficiently thick (∼30σ_CG_). A slow compression protocol was used for generating an equilibrated system of well-dispersed *stationary* MOF particles trapped at the air–water interface.^25^ Subsequently, equilibrium simulations of *freely mobile* MOFs were performed for ∼12 million timesteps for exploring their orientational and self-assembly behavior. The final orientation, *z*-position, and assembly morphology that the MOFs adopted were found to be insensitive to their initial orientation and position.

### Classification of Particle Orientations in Simulations

A method based on the interface-projected areas of MOF facets was used for classifying the MOFs into the three main orientational states: vertex-up, edge-up, and face-up (Figure S10).^25^ This involves determining the normal vector of each facet and using this vector to calculate the interface-projected areas of those facets pointing upward toward the air phase. From the total projected area, the % area contributed by the two most dominant faces, denoted *S*_1_ and *S*_2_, is obtained. If *S*_1_ < 37.5%, then the orientation is classified as “vertex-up”; otherwise, *S*_2_ is required to distinguish between the other two orientations. If *S*_1_ ≥ 0.375% and *S*_2_ ≥ 0.333%, the particle exhibits “face-up” orientation. If *S*_1_ ≥ 0.375% and *S*_2_ ≤ 0.333%, the particle exhibits “edge-up” orientation.

## Supporting Information Available

Synthetic details; tabulated data of molecular weight, grafting density, and brush height of all samples; chemical structure of CTAs and polymer-grafted MOFs; brush height calculation; images of MOF synthesis setup; images of photopolymerization setup; SEM images of all self-assembled materials; and description of coarse-grained simulation model, including Schemes S1–S7, Tables S1 and S2, and Figures S1–S12 (PDF)
Video S1, showing that removal of sections of the films formed from polymer-grafted MOFs with pMA was nearly impossible, with the entire film delaminating from the water surface (MP4)
