# Supplementary material for: Influence of Polymer Characteristics on the Self-Assembly of Polymer-Grafted Metal–Organic Framework Particles
Source: ACS Nano. 2022 Oct 17;16(11):18168–77. doi: 10.1021/acsnano.2c05175 (PMC9706656; doi:10.1021/acsnano.2c05175)
Supplement: Supplementary file 1 — nn2c05175_si_001.pdf [file nn2c05175_si_001.pdf]

Influence of Polymer Characteristics on the Self-Assembly of Polymer-Grafted MOF Particles

Kyle Barcus[†], Po-An Lin[‡], Yilong Zhou[‡], Gaurav Arya[‡], and Seth M. Cohen[†]*

[†]*Department of Chemistry and Biochemistry, University of California, San Diego, La Jolla, California 92093, United States*

[‡]*Department of Mechanical Engineering and Materials Science, Duke University, Durham, North Carolina 27710, United States*

SUPPORTING INFORMATION

Materials

All starting materials and reagents were purchased from Sigma-Aldrich and used without further purification unless otherwise specified. Dimethyl sulfoxide (DMSO, 99.7% extra dry) was purchased from Acros. Methyl methacrylate (MMA), methyl acrylate (MA), and benzyl methacrylate were purified by passing through a column filled with basic alumina (Sorbtech) to remove inhibitor and stored at 8 °C. Chain transfer agents (CTAs) 4-cyano-4-[(dodecylsulfanylthiocarbonyl)sulfanyl]pentanoic acid (CDSPA), 2-(dodecylthiocarbonothioylthio)-2-methylpropionic acid (DDMAT), and 4-cyano-4-(phenylcarbonothioylthio)pentanoic acid (CPADB) were purchased from Sigma-Aldrich and used as received. Blue LED light strips for the homemade photoreactor were purchased from ALITOVE, model 5050 Blue LED Flexible Strip Ribbon and powered using a 12V power adapter. The photoreactor was made from a thin-walled aluminum can (diameter = 16 cm) and the interior was wrapped with 50 cm of the LED strip (see Figure S4). Peristaltic pumps were purchased from New Era Pump Systems (Model 9000B).

Characterization

Nuclear Magnetic Resonance. Proton nuclear magnetic resonance spectra (^{1}H NMR) were recorded on a JEOL ECA 500 spectrometer (500 MHz). Chemical shifts are reported in parts per million (ppm) referenced to the appropriate solvent peak.

Gel Permeation Chromatography (GPC). Gel permeation chromatography (GPC) was performed in THF at 30 °C with a flow rate of 0.5 mL/min using an Agilent 1260 HPLC with diode array, Wyatt DAWN HELEOS 8+ multiangle laser light scattering detector

(MALS) with light wavelength at 690 nm, Viscostar III viscometer, and Optilab TrEX refractive index. Absolute molecular weights were determined using ASTRA software from Wyatt Technology.

Powder X-Ray Diffraction (PXRD). ~50 mg of dry MOF powder mounted on a silicon sample holder was used for analysis by PXRD. PXRD data was collected at ambient temperature on a Bruker D8 Advance diffractometer at 40 kV, 40 mA for Cu $K\alpha$ (l = 1.5418 Å), with a scan speed of 2 sec/step, a step size of 0.05° in 2θ, and a 2θ range of 2-50°.

BET Surface Area Analysis. Samples for analysis were evacuated on a vacuum line overnight at room temperature prior to analysis. ~50 mg samples were then transferred to pre-weighed sample tubes and degassed at 105 °C on a Micromeritics ASAP 2020 Adsorption Analyzer for a minimum of 12 h or until the outgas rate was <5 mmHg. After degassing, the sample tubes were re-weighed to obtain a consistent mass for the samples. BET surface area (m^2/g) measurements were collected at 77 K with N_2 on a Micromeritics ASAP 2020 Adsorption Analyzer using volumetric techniques.

Thermogravimetric Analysis (TGA). 1-5 mg of sample were placed in a 100 µL aluminum crucible. Samples were analyzed on a Mettler Toledo Star TGA/DSC using a temperature range of 30-600 °C scanning at 20 °C/min under an air atmosphere (75 cm^3/min flow rate) for sample degradation.

Scanning Electron Microscopy (SEM). MOF particles or monolayer films were transferred to silicon wafers on a sample holder disk and coated using an Ir-sputter coating for 12 seconds. A FEI Apreo SEM instrument was used for acquiring images using a 5 kV energy source under vacuum at a working distance at 10 mm.

Dynamic Light Scattering (DLS). A dilution of polymer-grafted MOFs (~ 0.1 mg/mL)

dispersed in 2 mL of toluene in a glass cuvette was analyzed at 23 °C with a Malvern Instruments Zetasizer Nano ZS90.

Synthesis of cat-CDSPA

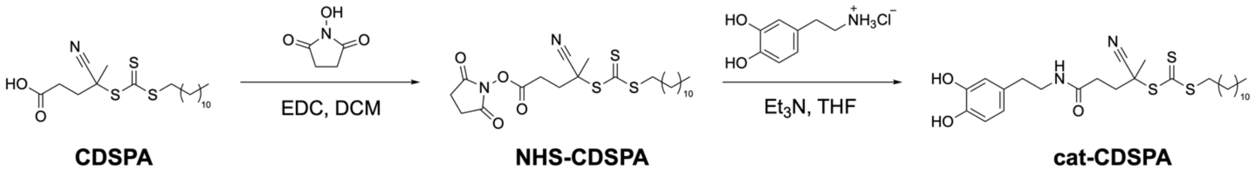

Scheme S1. Synthesis of catechol-ligated 4-cyano-4-[(dodecylsulfanylthiocarbonyl)sulfanyl]pentanoic acid (cat-CDSPA).

2,5-Dioxopyrrolidin-1-yl 4-cyano-4-(((dodecylthio)carbonothioyl)thio)pentanoate (NHS-CDSPA). A 100 mL round bottom flask was charged with 4-cyano-4-[(dodecylsulfanylthiocarbonyl)sulfanyl]pentanoic acid (CDSPA, Sigma-Aldrich), (1.00 g, 2.5 mmol, 1 eq.), *N*-hydroxysuccinimide (428 mg, 3.6 mmol, 1.5 eq.), and 40 mL of dry CH_2Cl_2 (Scheme S1). The flask was degassed with Ar for 5 min and cooled to 0 °C, after which 1-ethyl-3-(3-dimethylaminopropyl)carbodiimide (EDC) (760 mg, 4.0 mmol, 1.6 eq.) was added and the reaction was stirred at room temperature under an Ar atmosphere overnight. After stirring overnight, the organic layer was washed with 1 M HCl (1x100 mL), saturated $NaHCO_3$ (1x100 mL), brine, and dried with Na_2SO_4. The crude product was purified using column chromatography (EtOAc/hexane, 0-50%, elutes at 35%) to give a yellow solid. Yield: 1.01 g, 81%. ^{1}H NMR (300 MHz, $CDCl_3$): δ 3.38 – 3.27 (m, 2H), 2.93 (t, J = 8.4 Hz, 2H), 2.85 (s, 4H), 2.72 – 2.59 (m, 1H), 2.52 (ddd, J = 14.4, 9.3, 7.1 Hz, 1H), 1.88 (s, 3H), 1.76 – 1.63 (m, 2H), 1.58 (s, 1H), 1.42 (s, 1H), 1.26 (s, 18H), 0.88 (t, J = 6.7 Hz, 3H). ESI-MS(+) Experimental: *m/z* 523.13 $[M+Na]^+$, Calculated for $[C_{23}H_{36}N_2O_4S_3]$: 500.14.

2-Cyano-5-((3,4-dihydroxyphenethyl)amino)-5-oxopentan-2-yl dodecyl carbonotrithioate (cat-CDSPA). A 100 mL round bottom flask was charged with NHS-

CDSPA (1 g, 2.0 mmol, 1 eq.), dopamine hydrochloride (417 mg, 2.2 mmol, 1.1 eq.), and 50 mL of dry THF. The flask was degassed with Ar for 5 min and cooled to 0 °C, after which triethylamine (306 μl, 2.2 mmol, 1.1 eq.) was added dropwise and the reaction was left under an Ar atmosphere in an ice bath and allowed to warm to room temperature overnight. After 24 h, the reaction was diluted with 100 mL of 1M HCl and extracted with $CHCl_3$ (3x50 mL). The combined organic layers were washed with brine and dried with Na_2SO_4. The crude product was purified by column chromatography (EtOAc/hexane, 0-70%, elutes at 50%) and dried overnight in a vacuum oven to give an amber resin. Yield: 880 mg, 82%. ^{1}H NMR (500 MHz, $CDCl_3$): δ 6.81 (d, J = 8.0 Hz, 1H), 6.70 (d, J = 1.8 Hz, 1H), 6.59 (d, J = 8.0 Hz, 1H), 3.47 (dd, J = 5.9, 4.5 Hz, 2H), 3.32 (t, J = 7.5 Hz, 2H), 2.70 (t, J = 6.8 Hz, 2H), 2.54 – 2.45 (m, 1H), 2.45 – 2.38 (m, 2H), 2.37 – 2.28 (m, 1H), 1.86 (s, 3H), 1.75 – 1.60 (m, 3H), 1.43 – 1.33 (m, 2H), 1.25 (s, 18H), 0.88 (t, J = 6.9 Hz, 3H). ESI-MS(+) Experimental: *m/z* 539.21 $[M+H]^+$, Calculated for $[C_{27}H_{42}N_2O_3S_3]$: 538.24.

Synthesis of cat-DDMAT

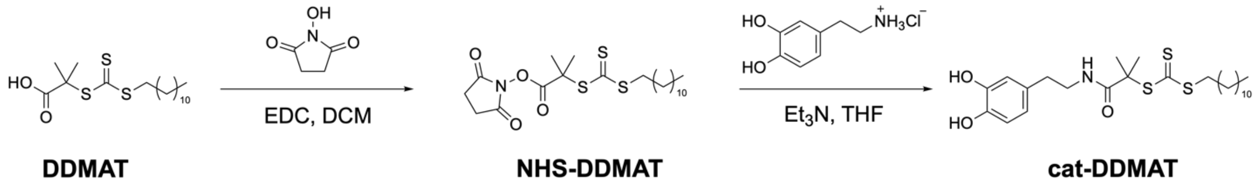

Scheme S2. Synthesis of catechol-ligated 1-((3,4-dihydroxyphenethyl)amino)-2-methyl-1-oxopropan-2-yl dodecyl carbonotrithioate (cat-DDMAT).

2,5-Dioxopyrrolidin-1-yl 2-methyl-2-(((dodecyllthio)carbonothioyl)thio)propanoate (NHS-DDMAT). A 100 mL round bottom flask was charged with 2-(((dodecylthio)carbonothioyl)thio)-2-methylpropanoic acid (DDMAT, Sigma-Aldrich) (1.00 g, 2.74 mmol, 1 eq.), *N*-hydroxysuccinimide (473 mg, 4.11 mmol, 1.5 eq.), and 40 mL of dry CH_2Cl_2 (Scheme S2). The flask was degassed with Ar for 5 min and cooled to 0 °C, after which 1-ethyl-3-(3-dimethylaminopropyl)carbodiimide (EDC) (841 mg, 4.39 mmol, 1.6 eq.) was added and the reaction was stirred at room temperature under an Ar atmosphere overnight. After stirring overnight, the organic layer was washed with 1 M HCl (1x100 mL), saturated $NaHCO_3$ (1x100 mL), brine, and dried with Na_2SO_4. The crude product was purified using column chromatography (EtOAc/hexane, 0-30%, elutes at 15%) to give yellow crystals. Yield: 1.1 g, 85%. ^{1}H NMR (500 MHz, $CDCl_3$): *δ* 3.33 (t, 2H), 2.85 (s, 4H), 1.88 (s, 6H) 1.77 – 1.62 (m, 2H), 1.42-1.20 (m, 18H), 0.88 (t, 3H). ESI-MS(+) Experimental: *m/z* 484.09 $[M+Na]^+$, Calculated for $[C_{25}H_{41}NO_3S_3]$: 461.17.

1-((3,4-Dihydroxyphenethyl)amino)-2-methyl-1-oxopropan-2-yl dodecyl carbonotrithioate (cat-DDMAT). A 100 mL round bottom flask was charged with NHS-DDMAT (1.00 g, 2.38 mmol, 1 eq.), dopamine hydrochloride (452 mg, 2.38 mmol, 1.1 eq.), and 50 mL of dry THF. The flask was degassed with Ar for 5 min and cooled to 0

°C, after which triethylamine (330 µl, 2.38 mmol, 1.1 eq.) was added dropwise and the reaction was left under an Ar atmosphere in an ice bath and allowed to warm to room temperature overnight. After 24 h, the reaction was diluted with 100 mL of 1M HCl and extracted with $CHCl_3$ (3x50 mL). The combined organic layers were washed with brine and dried with Na_2SO_4. The crude product was purified by column chromatography (EtOAc/hexane, 0-70%, elutes at 35%) and dried overnight in a vacuum oven to give an amber resin. Yield: 785 mg, 72%. ^{1}H NMR (500 MHz, $CDCl_3$): δ 6.81 (d, J = 8.0 Hz, 1H), 6.70 (s, 1H), 6.59 (d, J = 10.2 Hz, 1H), 3.53 – 3.42 (m, 2H), 3.32 (t, J = 7.4 Hz, 2H), 2.70 (t, J = 6.9 Hz, 2H), 1.86 (s, 6H), 1.68 (p, J = 7.4 Hz, 2H), 1.46-1.19 (m, 18H), 0.88 (t, J = 6.9 Hz, 3H). ESI-MS(+) Experimental: *m/z* 523.13 $[M+Na]^+$, Calculated for $[C_{25}H_{41}NO_3S_3]$: 499.22.

MOF Synthesis

UiO-66$_x$. UiO-66 was prepared using a continuous addition method as previously reported (*Angew. Chem. Int. Ed.* **2018**, 57, 7836-7840). The synthesis of UiO-66$_x$ (x = the particle edge length in nm measured by scanning electron microscopy, SEM) at 5 L scale was carried out at 120 °C under atmospheric pressure in DMF using formic acid as a modulator. Two separate 30 mM stock solutions were prepared in 5L jars. The terephthalic acid (H_2bdc) solution was prepared with 22.5 g of H_2bdc, 4.05 L of DMF, and 450 mL of formic acid, while the $ZrOCl_2{\cdot}8H_2O$ was prepared with 45 g of $ZrOCl_2{\cdot}8H_2O$ in 4.5 L of DMF. The reaction procedure is as follows. An initial 100 mL of the $ZrOCl_2{\cdot}8H_2O$ solution was added to a 5 L round bottom flask at 120 °C, then both the $ZrOCl_2{\cdot}8H_2O$ and H_2bdc stock solution were separately delivered by peristaltic pump with a feed rate of 12 mL/min for 5 min. The feed rate was accelerated to 32 mL/min for 55 minutes. After this first addition, 2.5 L of the reaction solution was removed from the reactor to obtain the first product, UiO-66$_{80}$, and then 1.5 L of metal stock solution and 1.5 L of ligand stock solution were further added into the remaining reaction solution at 30 mL/min for 50 min. Then 3 L of reaction solution was collected form the reactor to obtain the second product, UiO-66$_{120}$. Finally, 1.55 L of metal stock solution and 1.55 L of ligand stock solution were added into the reactor within 1 h at 25.8 mL/min, and the remaining reaction solution (3.7 L) was collected as the third product UiO-66$_{250}$. All products were first centrifuged (8000 rpm, 30-60 min) and washed with 40 mL DMF twice, and then solvent exchange was performed with by washing 3 times in 40 mL of methanol. The MOFs were left suspended in methanol at ~20 mg/mL until further use. Before any experiment, a fraction of the sample was removed and dried to determine the exact weight percent of the suspended

particles. For PXRD and N_2 sorption experiments the samples were dried in vacuum at 120 °C for 24 h.

Surface Functionalization of UiO-66 with cat-CDSPA

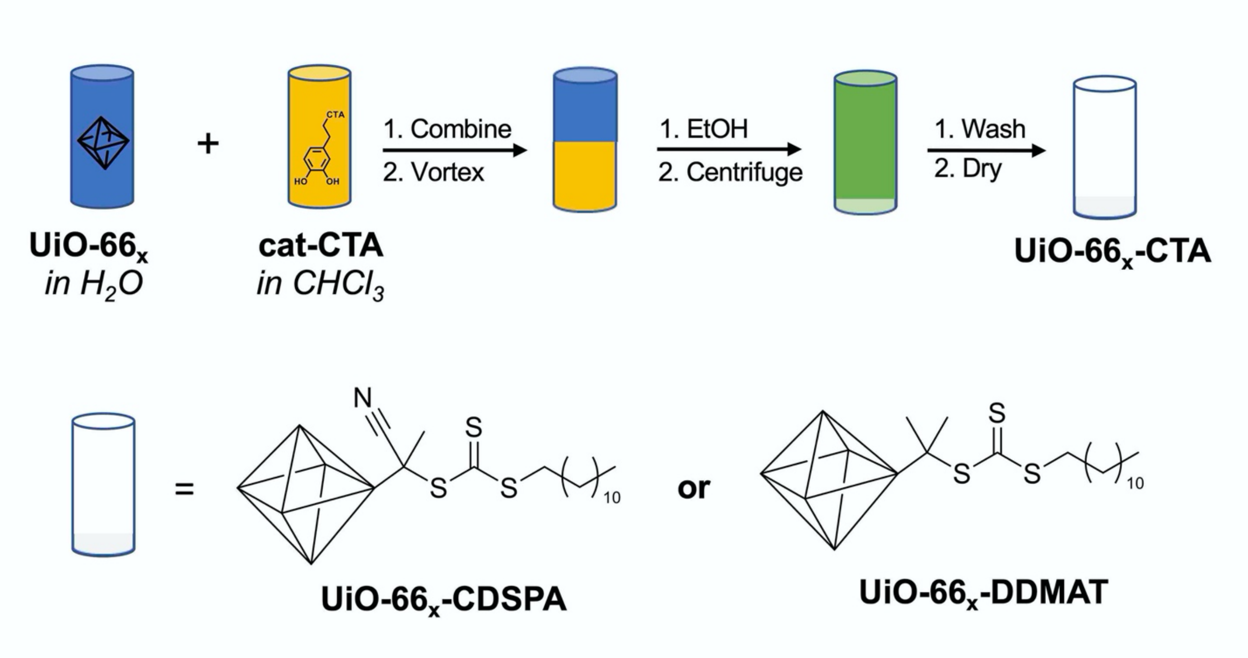

Scheme S3. Surface functionalization procedure of UiO-66x with either cat-CDSPA or cat-DDMAT. UiO-66_x is depicted by octahedron with aqueous and $CHCl_3$ layers represented by blue and orange colors, respectively. Mixed layers after ethanol addition is represented in green.

A 50 mL centrifuge tube was prepared with 200 mg of UiO-66_x in MeOH, centrifuged (8000 rpm, 15 min) to collect particles and redispersed in 10 mL of water. A separate vial was prepared with 10 mg of either cat-CDSPA or cat-DDMAT dissolved in 5 mL of $CHCl_3$ and added to the aqueous MOF suspension. The biphasic mixture was vortexed for 5 min, and then 20 mL of EtOH was added to form a homogeneous suspension. The

particles were collected by centrifugation (8000 rpm, 15 min), washed via repeated dispersion/centrifugation cycles with EtOH (2×25 mL, 30 min immersion each), followed by DMSO (3×20 mL, 30 min immersion each), and finally suspended in DMSO at a concentration of 80 mg/mL.

SI-PET-RAFT from UiO-66-CTA

General Polymerization Notes

All polymerizations were conducted using 40 mg of UiO-66_x-CTA regardless of particle size. The MOFs were stored as an 80 mg/mL suspension in DMSO; dried particles required extensive sonication to achieve adequate dispersion prior to polymerization. To ensure a controlled polymerization, it was necessary to add unbound CTA to the solution in excess to the surface (MOF) bound CTA. For each polymerization, the volume of monomer and solvent was held constant, and the targeted number of repeat units (degree of polymerization, DP) was adjusted by increasing or decreasing the amount of CTA relative to monomer while assuming 75% conversion. For example, if targeting a surface DP of 1500 for UiO-66_x-MA_{1500}, 1.68 mL (18.5 mmol) of methyl acrylate and 3.38 mg (9.3 μmol) of DDMAT are added, giving a molar ratio of MA:DDMAT 2000:1. For UiO-66_x-MA_{2400}, 1.68 mL (18.5 mmol) of methyl acrylate and 2.11 mg (5.80 μmol) of DDMAT are used giving a molar ratio of MA:DDMAT 3200:1. The amount of $Ir(ppy)_3$ was always set to $1x10^{-6}$ molar ratio relative to monomer. It is important to add the monomer to the reaction dropwise under rapid stirring, as it was observed that rapid addition of the monomer caused the MOF particles to irreversibly aggregate and settle out of the solution.

Polymerization of Methyl Acrylate from UiO-66-DDMAT

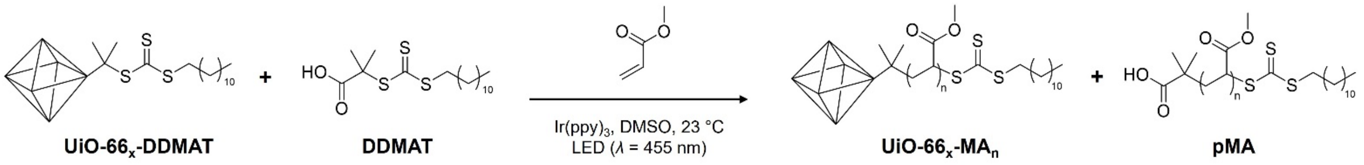

Scheme S4. Surface-initiated polymerization of methyl acrylate from UiO-66_x-DDMAT and the additional unbound DDMAT in solution.

UiO-66_x-MA_{1500}. A 10 mL round bottom flask was charged with a magnetic stir bar, 2.5 mL DMSO, and 500 µL of an 80 mg/mL stock solution of UiO-66_x-DDMAT suspended in DMSO. The solution was constantly stirred while DDMAT (3.38 mg, 9.3 µmol, 1 eq.) and $Ir(ppy)_3$ (12.1 µg, 0.018 µmol, .002 eq.) were added (from 10 mg/mL and 1 mg/mL DMF stock solutions, respectively). Methyl acrylate (1.68 mL, 18.5 mmol, 2000 eq.) was then added dropwise after which the suspension was left without stirring for 5 min to ensure that the MOF particles had not aggregated and settled. The reaction was then sealed tight with a rubber septum secured with a copper wire and degassed with Ar for 30 min. The reaction mixture was transferred to a homemade blue light photoreactor and irradiated until mixture could no longer stir.

Polymerization of Methyl Methacrylate from UiO-66-CDSPA

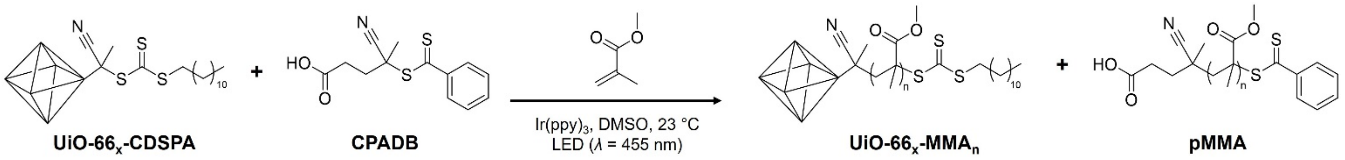

Scheme S5. Surface-initiated polymerization of methyl methacrylate from UiO-66x-CDSPA and the additional unbound CPADB in solution.

UiO-66$_x$-MMA$_{1500}$. A 10 mL round bottom flask was charged with a magnetic stir bar, 2.0 mL DMSO, and 500 µL of an 80 mg/mL stock solution of UiO-66$_x$-CDSPA suspended in DMSO. The solution was constantly stirred while CPADB (2.60 mg, 9.3 µmol, 1 eq.) and Ir(ppy)$_3$ (12.1 µg, 0.018 µmol, .002 eq.) were added (from 10 mg/mL and 1 mg/mL DMF stock solutions, respectively). Methyl methacrylate (1.98 mL, 18.5 mmol, 2000 eq.) was then added dropwise after which the suspension was left without stirring for 5 min to ensure that the MOF particles had not aggregated and settled. The reaction was then sealed tight with a rubber septum secured with a copper wire and degassed with Ar for 30 min. The reaction mixture was transferred to a homemade blue light photoreactor and irradiated until mixture could no longer stir.

Polymerization of Benzyl Methacrylate from UiO-66-CDSPA

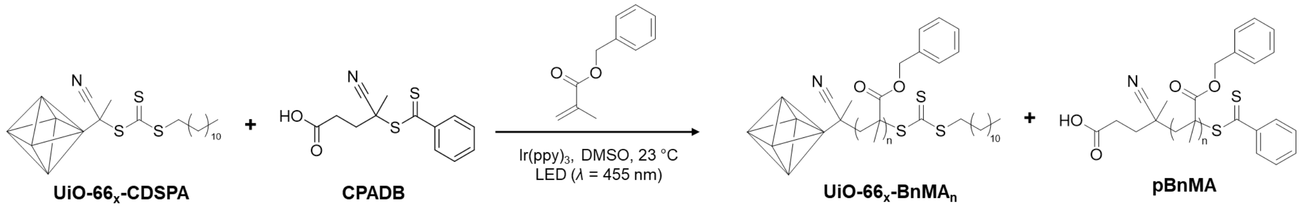

Scheme S6. Surface-initiated polymerization of benzyl methacrylate from UiO-66x-CDSPA and the additional unbound CPADB in solution.

UiO-66$_x$-BnMA$_{1500}$. A 10 mL round bottom flask was charged with a magnetic stir bar, 1.5 mL DMSO, and 500 µL of an 80 mg/mL stock solution of UiO-66$_x$-CDSPA suspended in DMSO. The solution was constantly stirred while CPADB (1.63 mg, 5.84 µmol, 1 eq.) and Ir(ppy)$_3$ (7.65 µg, 0.012 µmol, .002 eq.) were added (from 10 mg/mL and 1 mg/mL DMF stock solutions, respectively). Benzyl methacrylate (1.98 mL, 11.7 mmol, 2000 eq.) was then added dropwise after which the suspension was left without stirring for 5 min to ensure that the MOF particles had not aggregated and settled. The reaction was then sealed tight with a rubber septum secured with a copper wire and degassed with Ar for 30 min. The reaction mixture was transferred to a homemade blue light photoreactor and irradiated until mixture could no longer stir.

General Workup. The rubber septa was removed, and a small sample of the reaction was collected and diluted in 700 µL $CDCl_3$ for ^{1}H NMR analysis to determine conversion. The primary reaction mixture was diluted with 40 mL THF, transferred to a 50 mL centrifuge tube, and the particles were collected by centrifugation until the supernatant was no longer cloudy (UiO-66$_{250}$: 8000 rpm, 20 min; UiO-66$_{120}$: 8000 rpm, 30 min; UiO-

66_{80}: 10000 rpm, 30 min). The MOF particles were then washed by redispersing in 5×40 mL of THF (20 min immersion after redispersing per wash cycle, followed by centrifugation under conditions specified above) to ensure complete removal of any unbound, free polymer. Gel permeation chromatography (GPC) of the supernatant after last wash was used to confirm removal of all free polymer. The washed MOF particles were resuspended in 10 mL of THF and transferred to a 15 mL centrifuge tube before dividing into samples for further for analysis and self-assembly experiments. For all materials, several samples were prepared for analysis and experiments (Scheme 1, main text). From the THF dispersion, 2 mL of solution were transferred to a 15 mL centrifuge tube and exchanged with 3×10 mL CH_2Cl_2 (8000 rpm, 20 min) for thermogravimetric analysis (TGA). The remaining 8 mL of the THF dispersion were then exchanged with 3×10 mL of toluene (8000 rpm, 10 min) and 1 mL was removed for GPC digestion analysis and another 1 mL removed for dynamic light scattering (DLS) analysis. The remaining ~6 mL of particle solution was used for self-assembly at an air-water interface.

Removal of Free-Standing Area of SAMM on Wire Loop

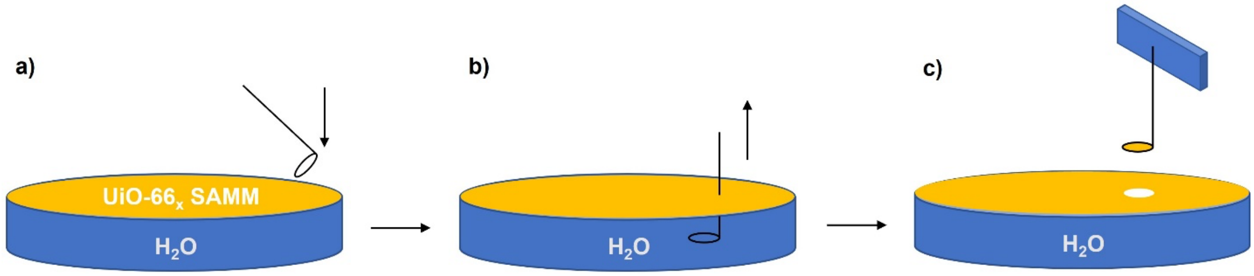

Scheme S7. A segment of the monolayer is removed using a copper wire loop by a) inserting the wire loop under the surface of the film, b) gently lifting the loop from underneath the film parallel to the water surface, and c) carefully hanging the suspended film to let the water dry. A successful free-standing monolayer was identified by a dried film that remained suspended across the wire loop.

Supporting Figures and Tables

Table S1. Synthesis conditions and characteristics of polymer grafted MOF nanoparticles prepared in this study ($Đ = M_w/M_n$, N = degree of polymerization, h = calculated brush height, σ = grafting density).

Sample	Monomer Eq.	$Ir(ppy)_3$ Eq.	Mn (kDa)	Mw (kDa)	Đ	*N*	Size DLS (nm)	*h* (nm)	Polymer wt. %	*σ* (chain/nm^2)
**UiO-66$_{80}$-MMA$_{520}$**	1200	0.001	52.0	69.2	1.33	520	124	17	26	0.26
**UiO-66$_{80}$-MMA$_{1186}$**	1500	0.0015	118.6	140.8	1.19	1186	175.9	43	23	0.10
**UiO-66$_{80}$-MMA$_{1453}$**	2000	0.002	145.3	182.1	1.25	1453	168	39	31	0.12
**UiO-66$_{80}$-MMA$_{3195}$**	4000	0.004	319.5	463	1.45	3195	347.6	129	46	0.10
**UiO-66$_{80}$-MA$_{1574}$**	2000	0.002	135.4	145.7	1.08	1574	245.9	78	78	1.02
**UiO-66$_{80}$-MA$_{2279}$**	2500	0.0025	196.0	218.4	1.12	2279	314.9	113	67	0.40
**UiO-66$_{80}$-MA$_{2747}$**	3500	0.0035	236.2	291.4	1.23	2747	257	84	23	0.05
**UiO-66$_{80}$-BnMA$_{715}$**	1500	0.0015	125.9	239.2	1.9	715	193.2	52	31	0.13
**UiO-66$_{80}$-BnMA$_{1855}$**	2500	0.0025	326.4	422.4	1.29	1855	182	46	32	0.06
**UiO-66$_{80}$-BnMA$_{2151}$**	3000	0.003	378.5	527.5	1.39	2151	187.8	49	32	0.05
**UiO-66$_{120}$-MMA$_{452}$**	1000	0.001	45.0	63.8	1.41	452	157.2	12	21	0.23
**UiO-66$_{120}$-MMA$_{1180}$**	1500	0.0015	118.0	145.7	1.22	1180	221.3	44	18	0.07
**UiO-66$_{120}$-MMA$_{1952}$**	2500	0.0025	195.2	249.6	1.28	1952	297.6	82	31	0.09
**UiO-66$_{120}$-MMA$_{2539}$**	3000	0.003	253.9	331.5	1.31	2539	357.9	112	33	0.08
**UiO-66$_{120}$-MMA$_{4071}$**	4500	0.0045	407.1	657.3	1.62	4071	478	172	41	0.07
**UiO-66$_{120}$-MA$_{452}$**	1500	0.001	38.9	40.2	1.03	452	198.1	32	19	0.23
**UiO-66$_{120}$-MA$_{1294}$**	1500	0.0015	111.3	122.6	1.1	1294	244	55	41	0.24
**UiO-66$_{120}$-MA$_{2056}$**	2500	0.0025	176.8	190.7	1.08	2056	273	70	43	0.17
**UiO-66$_{120}$-MA$_{2370}$**	3500	0.0035	203.8	219	1.08	2370	293	80	68	0.40
**UiO-66$_{120}$-BnMA$_{1054}$**	1500	0.0015	185.5	3157	1.7	1054	242.5	54	25	0.07
**UiO-66$_{120}$-BnMA$_{2878}$**	3000	0.003	506.6	537.3	1.06	2878	250.5	58	23	0.02
**UiO-66$_{250}$-MMA$_{690}$**	1500	0.0015	69.0	77.5	1.12	690	313.9	18	18	0.12
**UiO-66$_{250}$-MMA$_{1460}$**	2000	0.002	146.0	187.2	1.28	1460	318	20	17	0.05
**UiO-66$_{250}$-MMA$_{2566}$**	3000	0.003	256.6	318.4	1.24	2566	350.7	36	19	0.03
**UiO-66$_{250}$-MMA$_{4552}$**	5000	0.005	455.2	597.2	1.31	4552	374.3	48	23	0.02

**UiO-66$_{250}$-MA$_{750}$**	1500	0.0015	64.5	66.4	1.03	750	332	27	12	0.08
**UiO-66$_{250}$-MA$_{994}$**	2000	0.002	85.5	90.7	1.06	994	329.9	26	14	0.07
**UiO-66$_{250}$-MA$_{2429}$**	3000	0.003	208.9	222.3	1.06	2429	397.9	60	28	0.07
**UiO-66$_{250}$-MA$_{2588}$**	3000	0.003	222.6	237.5	1.07	2588	410.6	66	27	0.07
**UiO-66$_{250}$-BnMA$_{1309}$**	1500	0.0015	230.4	271	1.43	1309	335.9	29	19	0.04
**UiO-66$_{250}$-BnMA$_{2920}$**	3500	0.0035	513.9	542	1.06	2920	326.5	24	17	0.02

Table S2. Molecular weight values of free polymer with: no MOF present, with unfunctionalized UiO-66$_{120}$, and UiO-66$_{120}$-DMeCTA. Equivalents of monomer and photocatalyst are listed.

Sample	Monomer	Ir(ppy)$_3$	% Conversion	M_n (g/mol)	M_w (g/mol)	Đ
No MOF	1500	0.0015	89	117,000	128,000	1.09
UiO-66$_{120}$	1500	0.0015	84	95,100	119,400	1.255
UiO-66$_{120}$-cat-DDMAT	1500	0.0015	81	90,400	110,200	1.220

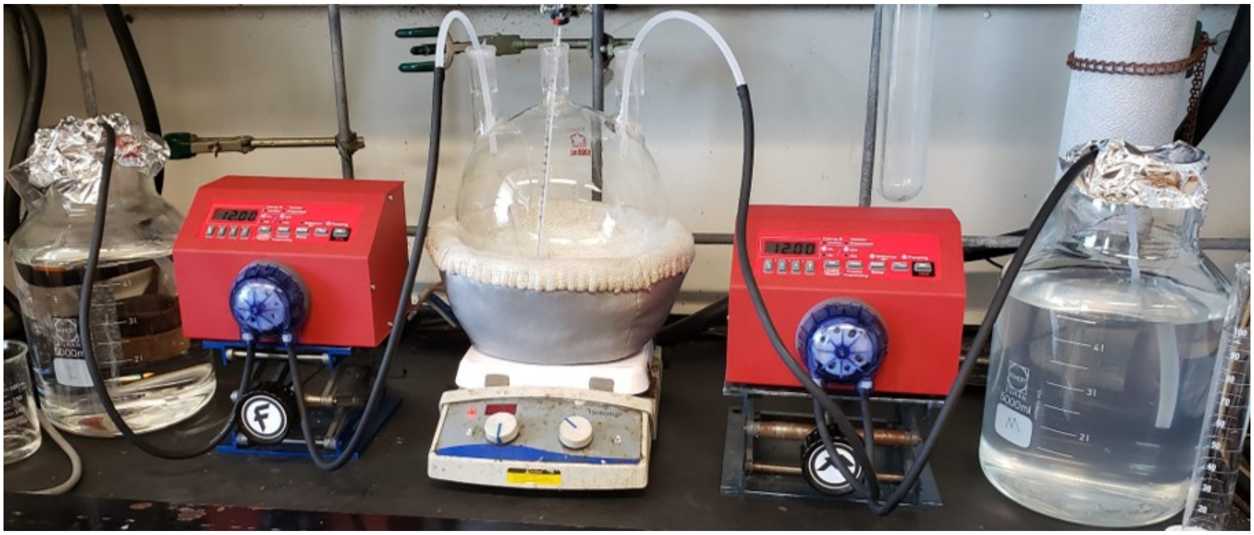

Figure S1. Experimental setup for the large- scale synthesis of UiO-66_x. 4L Flasks (left and right sides) contain separate stock solutions of H_2bdc and $ZrOCl_2{\cdot}8H_2O$ in DMF (30 mM). Peristaltic pumps deliver both solutions at prescribed feed rates to the 4L 3-neck round bottom flask set in a heating mantel. The mantel was used to hold the temperature of the reaction solution at 120 °C.

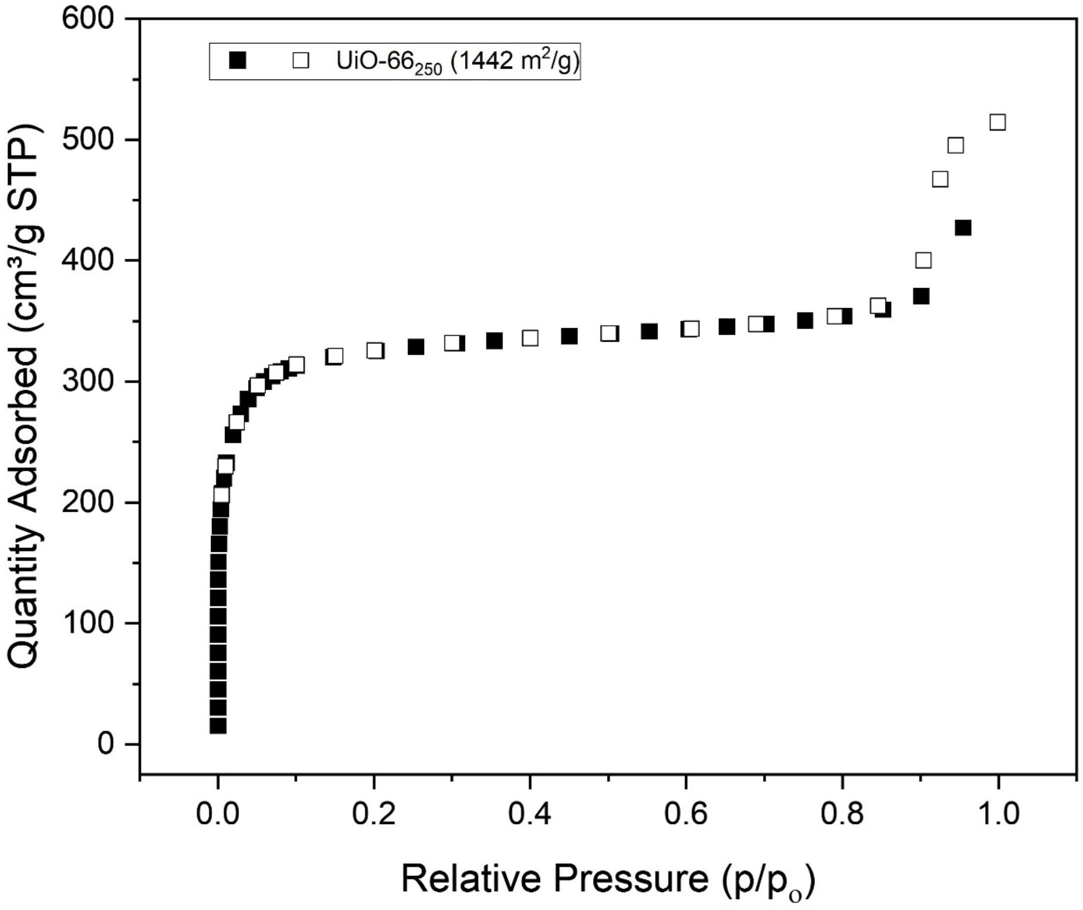

Figure S2. N_2 sorption isotherm for UiO-66_{250} with respective BET surface area.

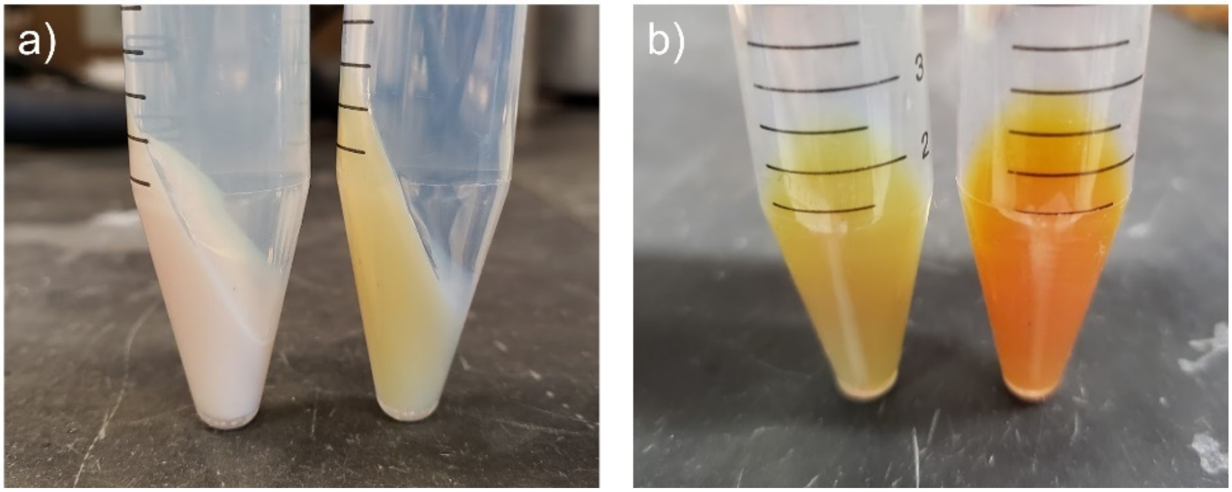

Figure S3. a) UiO-66$_{120}$ before (*left*) and after (*right*) functionalization with cat-CDSPA. b) UiO-66$_{80}$ functionalized with cat-CDSPA (*left*) and cat-DDMAT (*right*).

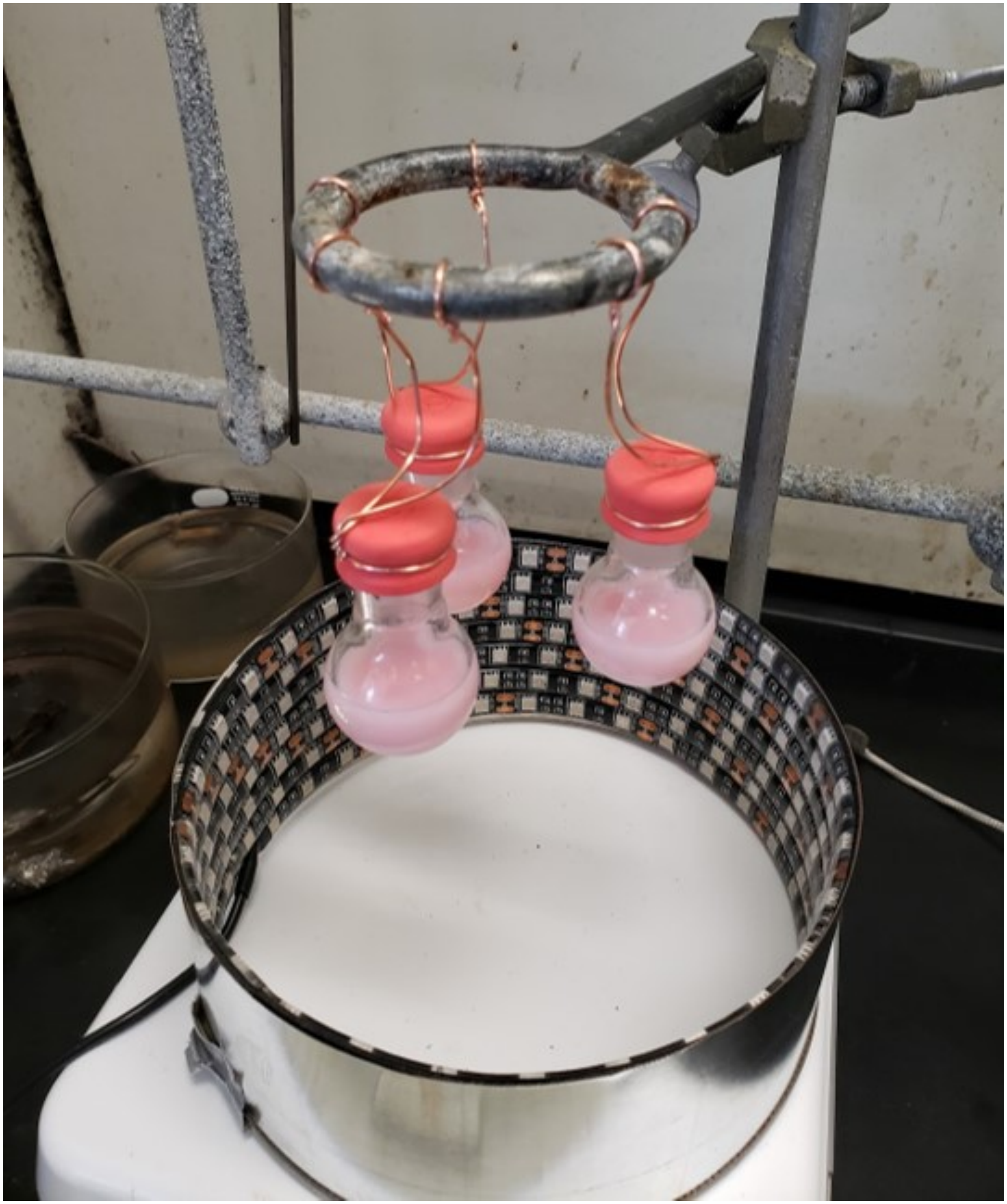

Figure S4. Home built LED reaction vessel lined with blue led strips.

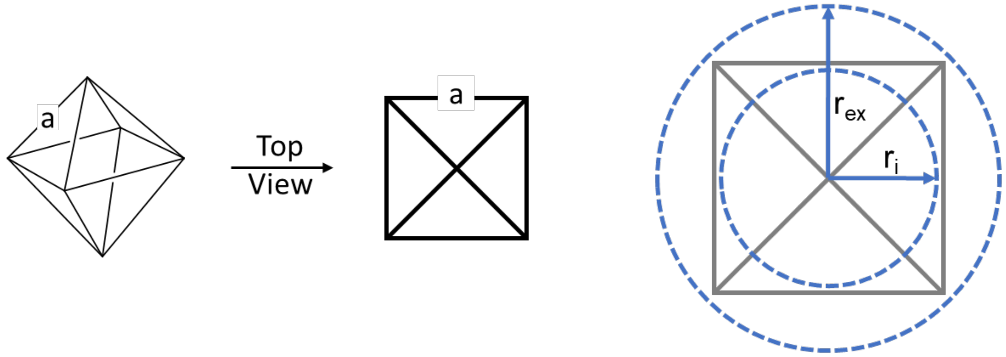

$$r_{\mathrm{i}} = \frac{a}{6}\sqrt{6}$$

$$r_{\mathrm{ex}} = \frac{a}{2}\sqrt{2}$$

$$r_{\mathrm{o}} = \frac{r_{\mathrm{i}} + r_{\mathrm{ex}}}{2}$$

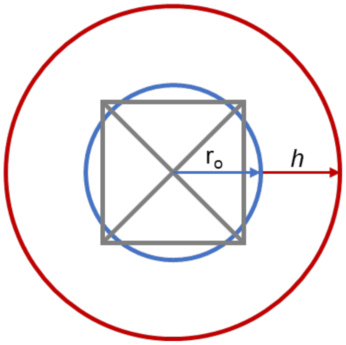

$$h = r - r_{\mathrm{o}}$$

$$h = \frac{d_{\mathrm{DLS}}}{2} - \frac{\frac{a}{6}\sqrt{6} + \frac{a}{2}\sqrt{2}}{2}$$

Figure S5. Polymer brush height determination using an intermediate size sphere as an internal radius. DLS of polymer grafted MOF nanoparticles is based on the diameter of a sphere with the same diffusion constant as the particle in solution; therefore, a simplified model of the octahedral MOF was used to determine brush height. A sphere inside the MOF which contacts each face tangentially has a radius r_i = (a/6)*6^{-1} and a sphere outside the octahedron in contact with each vertex has a radius r_{ex} = (a/2)*2^{-1}. The average of these two radii, termed r_o, was used to represent a sphere of average distance from the center of the MOF particle. The brush height was then determined by subtracting r_o from the radius given by DLS.

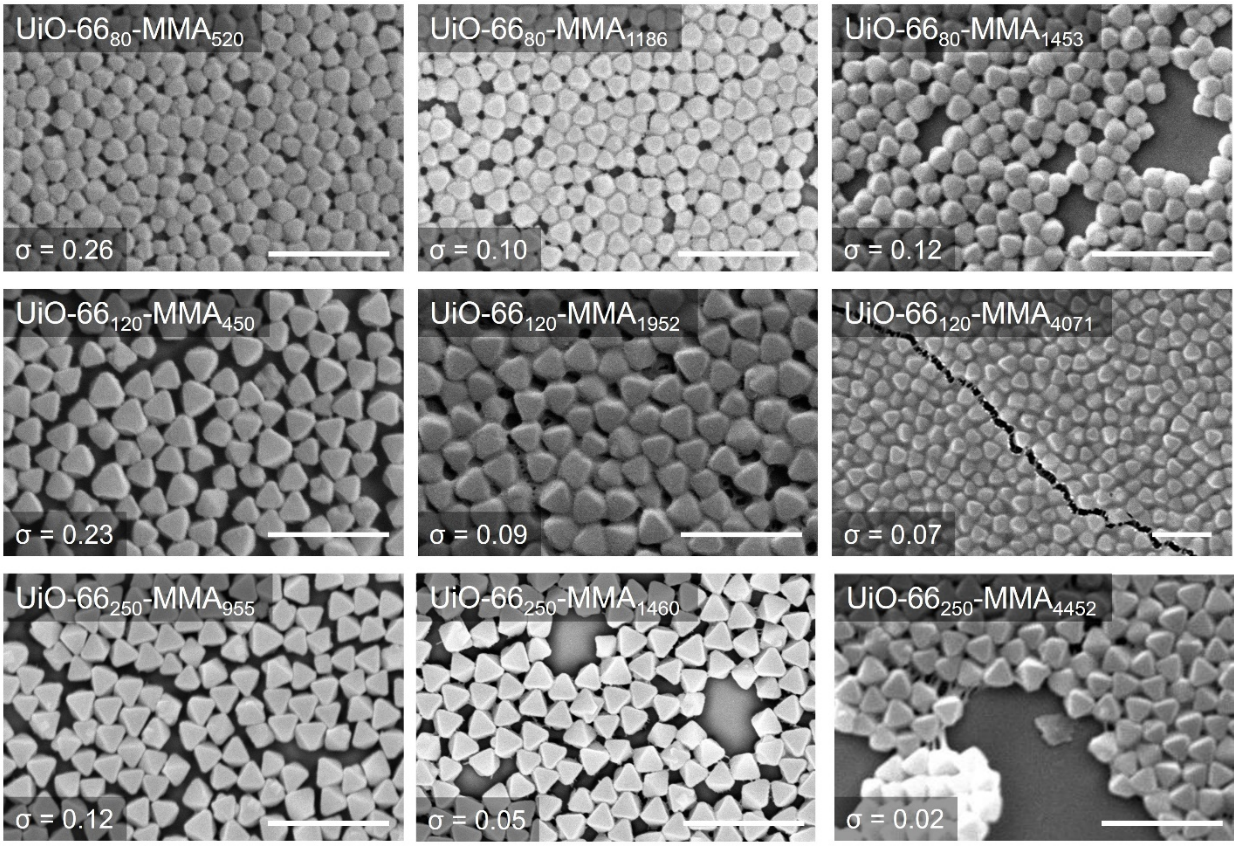

Figure S6. SEM images of self-assembled films of UiO-66$_x$-MMA$_n$. Grafting density values are shown (σ). Scale bars are 500 nm for the first two rows and 1 μm for the bottom row.

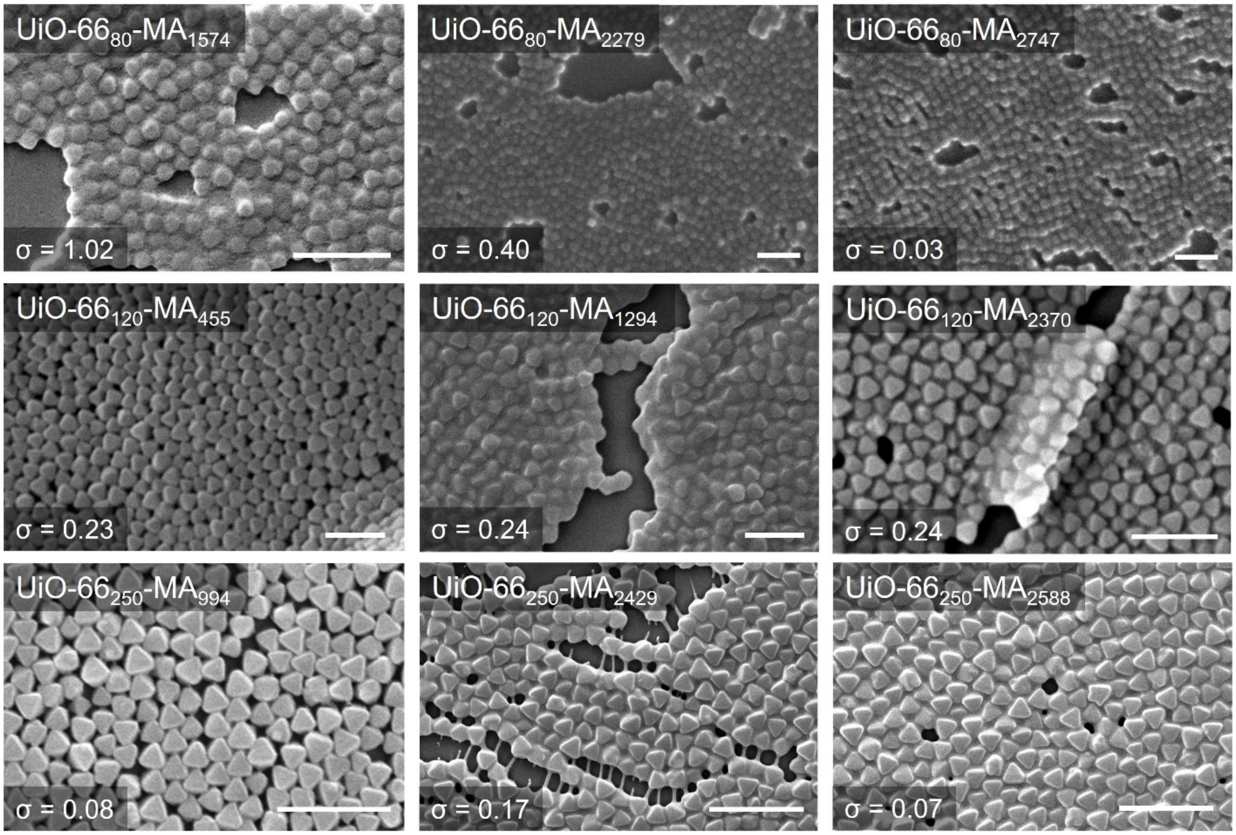

Figure S7. SEM images of self-assembled films of UiO-66_x-MA_n. Grafting density values are shown (σ). Scale bars are 500 nm for the first two rows and 1 μm for the bottom row.

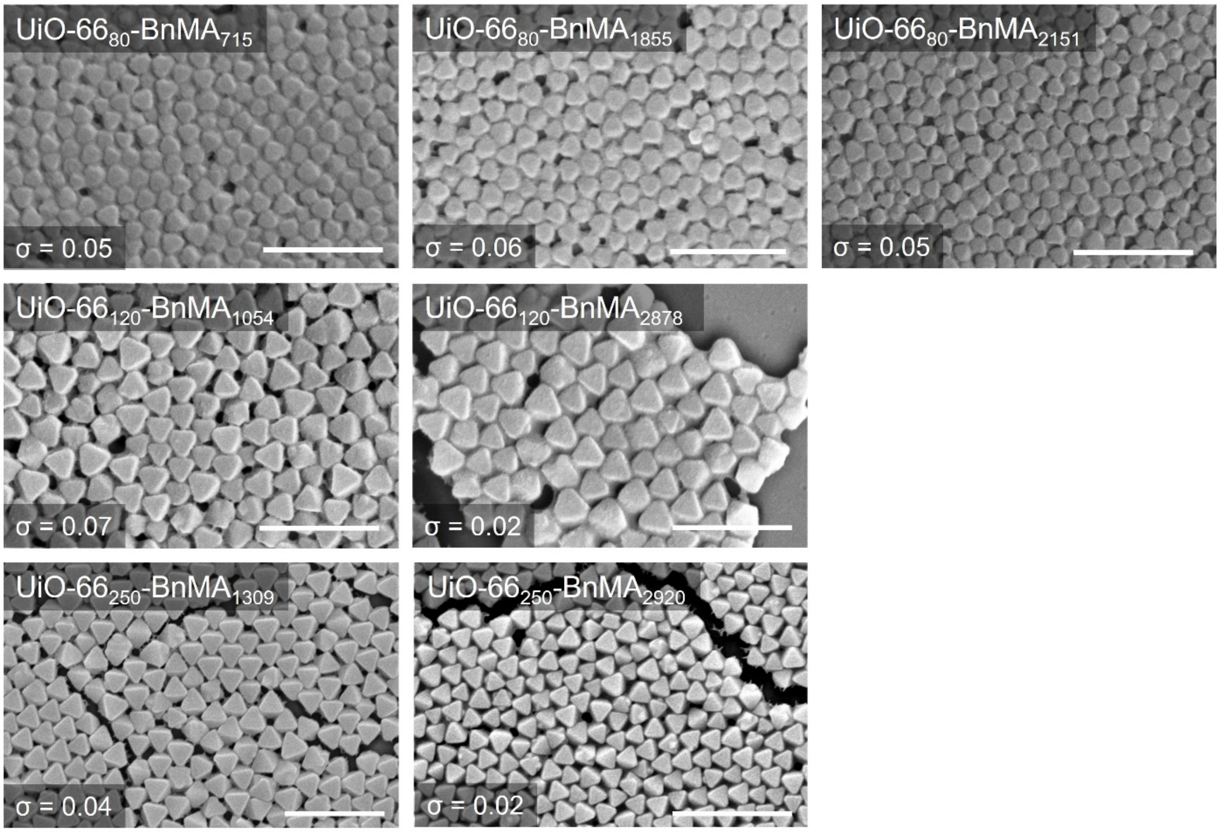

Figure S8. SEM images of self-assembled films of UiO-66_x-$BnMA_n$. Grafting density values are shown (σ). Scale bars are 500 nm for the first two rows and 1 μm for the bottom row.

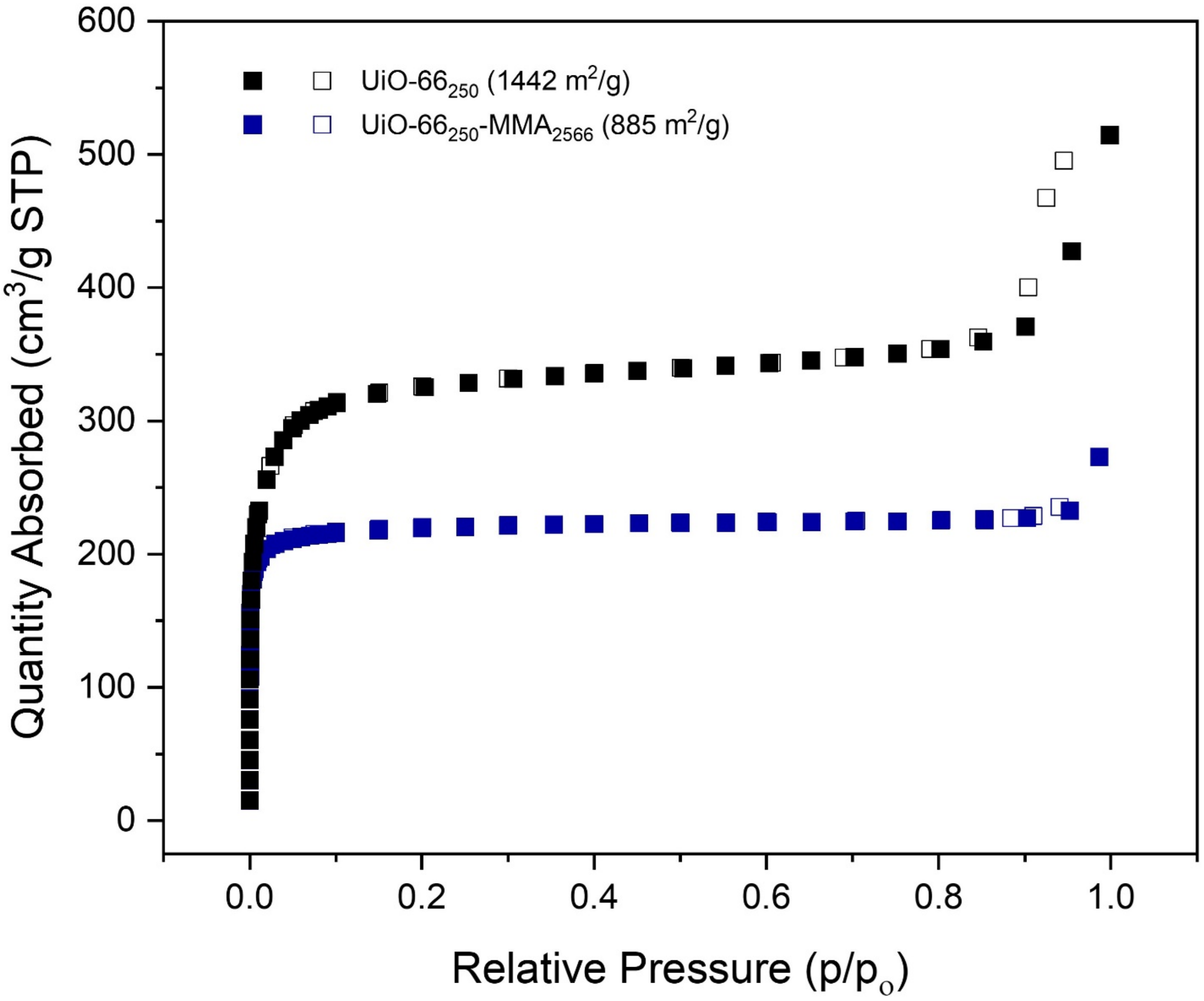

Figure S9. N_2 sorption isotherm for UiO-66_{250} (black) and UiO-66_{250}-MMA_{2566} (blue) with respective BET surface areas shown.

Coarse-Grained Model

To investigate the dynamics of polymer-grafted MOFs at an air-water interface, we extended the coarse-grained model we previously developed for studying the assembly of polymer-grafted nanocubes at polymer interfaces. The MOFs were modeled as rigid bodies of regular octahedra geometry constructed out of coarse-grained beads, each of size σ_{CG}, where σ_{CG} corresponds to a length scale of ~10 nm. Three sets of MOFs were constructed with edge lengths of $L_{\mathrm{MOF}} = 9\sigma_{CG}$, $13\sigma_{CG}$, and $28\sigma_{CG}$ corresponding to the experimental MOFs with edge lengths 80, 120, and 250 nm.

Polymer grafts (denoted by "g") were attached to all eight facets of the MOFs at a grafting density of 0.3 chains/${\sigma_{CG}}^2$ to target the low grafting density regimes investigated in experiments. The grafts were treated as Kremer-Grest bead-chains, where short segments of the chain are described by coarse-grained beads of size σ_{CG} and mass m. We explored chain lengths of $L_{\mathrm{g}} = 1, 2, 4,$ and 6 beads to characterize the effect of degree of polymerization studied in the experiments. Adjacent beads in the chains, denoting *bonded segments*, interacted with each other through finitely extensible nonlinear elastic (FENE) spring and Weeks-Chandler-Anderson (WCA) potentials. The FENE spring potential, which ensures that bonded segments do not stretch beyond a cutoff distance, is given by:

$$U_{\mathrm{FENE}}(r; k, R_0) = -\frac{k}{2} R_0^2 \ln\left[1 - \left(\frac{r}{R_0}\right)^2\right] \quad (1)$$

where r is the separation distance between the segments, $k = 30\varepsilon/{\sigma_{CG}}^2$ is the spring constant, ε is the characteristic energy parameter, and $R_0 = 1.5\sigma_{CG}$ is the maximum possible length of the spring. The WCA potential, a short-range purely repulsive potential

that models excluded-volume interactions between the bonded segments, can be conveniently presented in the form of a cut-and-shifted Lennard-Jones (LJ) potential:

$$U_{\mathrm{LJ}}(r;\sigma_{CG},\varepsilon,r_{\mathrm{c}}) = \begin{cases} 4\varepsilon\left[\left(\frac{\sigma_{CG}}{r}\right)^{12} - \left(\frac{\sigma_{CG}}{r}\right)^{6} - \left(\frac{\sigma_{CG}}{r_c}\right)^{12} + \left(\frac{\sigma_{CG}}{r_c}\right)^{6}\right] & r < r_c \\ 0 & r \geq r_c \end{cases} \quad (2)$$

with a cutoff distance of $r_c = 2^{1/6}\sigma_{CG}$. The grafts were also attached to the surface beads of the MOFs via the combined FENE-WCA potential.

The water (w) and air (a) phases were also treated using coarse-grained beads. The water beads interact with each other via an attractive Lennard-Jones (LJ) potential $U_{\text{w-w}} = U_{\mathrm{LJ}}(r;\sigma_{CG},\varepsilon,r_{\mathrm{c}} = 2.5\sigma_{CG})$, which accounts for both attractive and the excluded-volume interactions due to the larger cutoff of $r_c = 2.5\sigma_{CG}$. The air beads interact with each other and with water beads *via* the excluded-volume WCA potential $U_{\text{a-a}} = U_{\text{w-a}} = U_{\mathrm{LJ}}(r;\sigma_{CG},\varepsilon,r_{\mathrm{c}} = 2^{1/6}\sigma_{CG})$. The two fluids were maintained at number densities of 0.4 and 0.02 beads/σ_{CG}^3, which together with the above interaction potentials led to stable liquid- and gas-like phases and a sufficiently large surface tension between them at the simulated temperature of $0.7\varepsilon/k_B$.

Pairs of *non-bonded* segments within a polymer chain or across chains interacted with each other *via* the LJ potential $U_{\mathrm{g-g}} = U_{\mathrm{LJ}}(r;\sigma_{CG},\varepsilon,r_c = 2.5\sigma_{CG})$. The interactions between the grafts and the air phase were also treated by the LJ potential $U_{\mathrm{g-a}} = U_{\mathrm{LJ}}(r;\sigma_{CG},\varepsilon,r_{\mathrm{c}} = 2.5\sigma_{CG})$. Depending on the hydrophobicity of the grafts being studied, the grafts were either partially miscible or fully miscible with the water phase: when partially miscible, the segments interacted *via* the LJ potential $U_{\text{g-w}} = U_{\mathrm{LJ}}(r;\sigma_{CG},\lambda\varepsilon,r_{\mathrm{c}} = 2.5\sigma_{CG})$ with a reduced attraction strength of $\lambda\varepsilon$,

where $0 < \lambda < 1$; and when fully miscible (strongly hydrophilic), they interacted *via* the original LJ potential $U_{\text{g-w}} = U_{\text{LJ}}(r; \sigma_{CG}, \varepsilon, r_{\text{c}} = 2.5\sigma_{CG})$. To account for the hydrophilic nature of the studied MOFs, the interactions between the MOFs and water beads were treated by the LJ potential $U_{\text{MOF-w}} = U_{\text{LJ}}(r; \sigma_{CG}, \varepsilon, r_{\text{c}} = 2.5\sigma_{CG})$. The interactions between the MOFs, the MOFs and air beads, and the MOFs and polymer grafts were all treated using an excluded-volume WCA potential $U_{\text{MOF-MOF}} = U_{\text{MOF-a}} = U_{\text{MOF-g}} = U_{\text{LJ}}\left(r; \sigma_{CG}, \varepsilon, r_{\text{c}} = 2^{1/6}\sigma_{CG}\right)$.

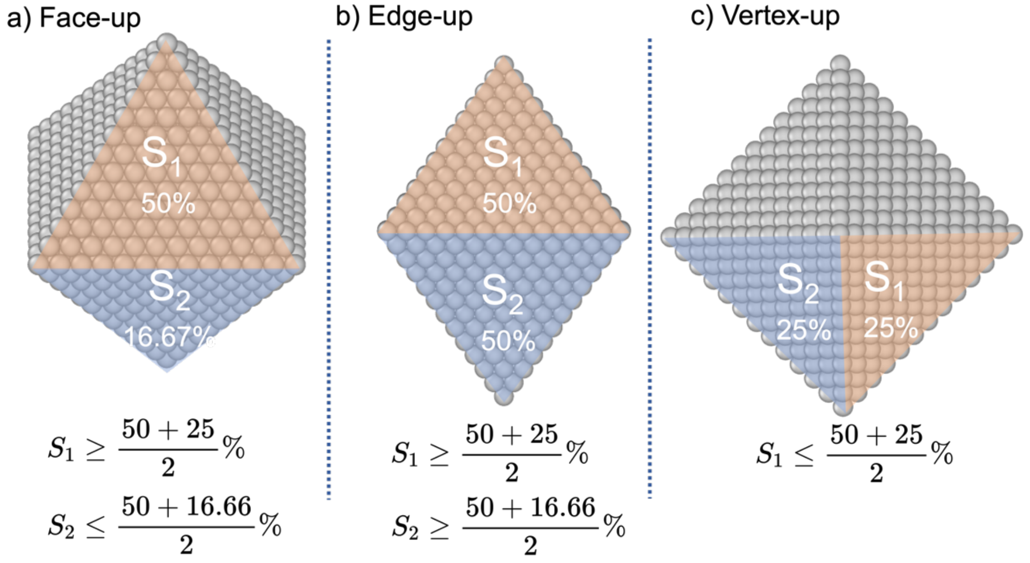

Figure S10. Orientation classification method. S_1 and S_2 represent the % of the total interface-projected area contributed by the first- and second-most dominant facets of the octahedral MOF particle. (a) If $S_1 \geq 0.375\%$ and $S_2 \geq 0.333\%$, the particle exhibits 'face-up' orientation. (b) If $S_1 \geq 0.375\%$ and $S_2 \leq 0.333\%$, the particle exhibits 'edge-up' orientation. (c) If $S_1 \leq 0.375\%$, the particle exhibits 'vertex-up' orientation.

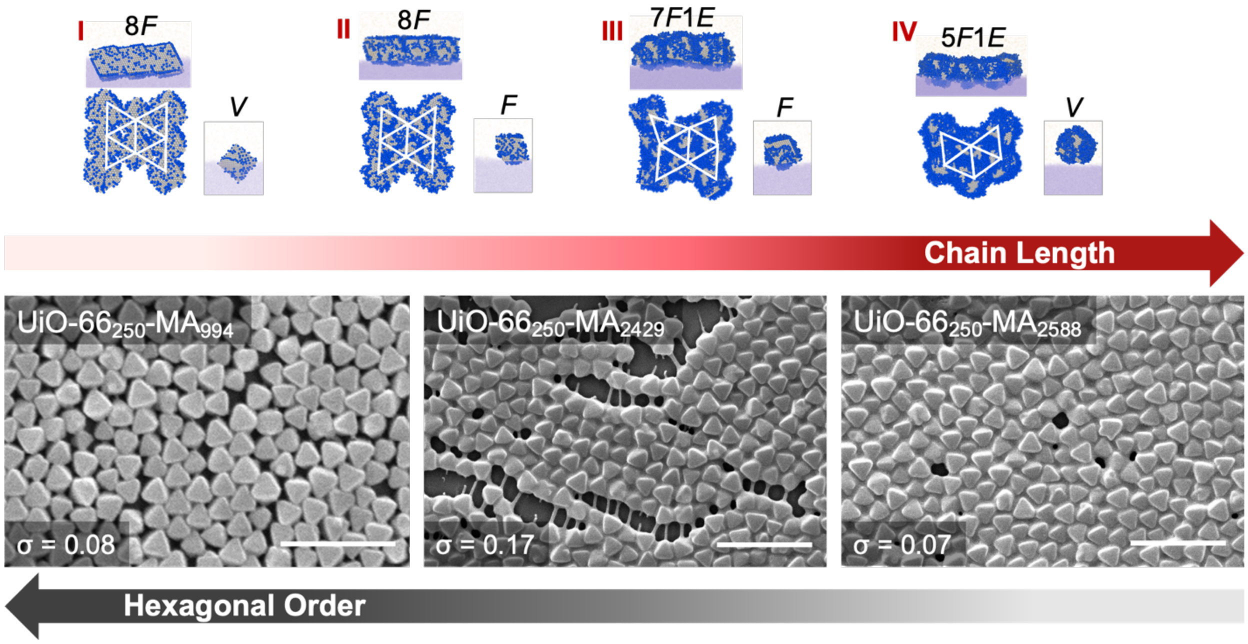

Figure S11. Comparison of simulation and experimental self-assembly showing decreasing hexagonal order with increasing polymer length. Side and top view of structures assembled with MOFs of increasing: graft lengths $L_g = 1\sigma_{CG},\ 2\sigma_{CG},\ 4\sigma_{CG}$, and $6\sigma_{CG}$ for fixed MOF size $L_{MOF} = 13\sigma_{CG}$ and graft hydrophilicity $\lambda = 0.2$ (*top panel*); SEM images of self-assembled films of UiO-66_{250}-MA_n, scale bars are 1 μm (*bottom panel*).

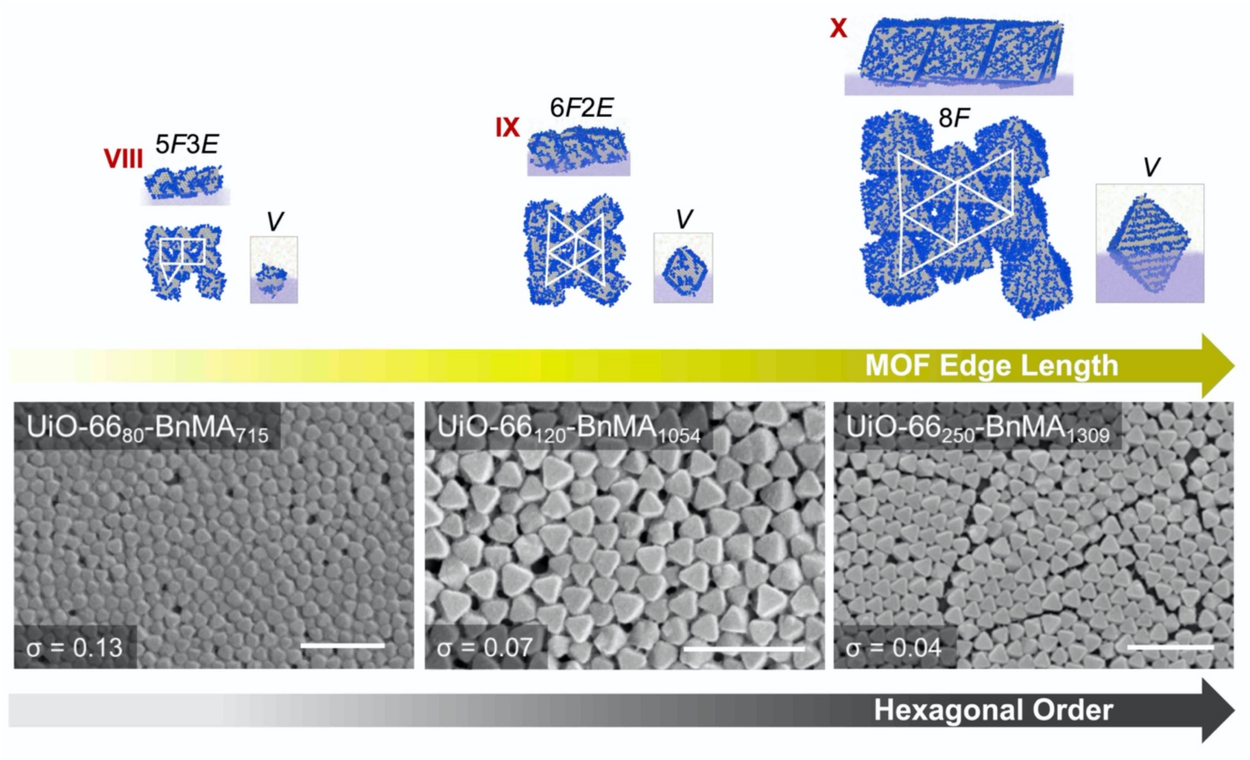

Figure S12. Comparison of simulation and experimental self-assembly showing increasing hexagonal order with increasing particle size. Side and top view of structures assembled with MOFs of increasing edge length $L_{MOF} = 9\sigma_{CG}, 13\sigma_{CG}$, and $28\sigma_{CG}$ for fixed $L_g = 2\sigma_{CG}$, $\lambda = 0.4$ (*top panel*). Insets show the orientation adopted by a single, isolated MOF particle. SEM images of self-assembled films of UiO-66_x-$BnMA_n$, scale bars are 500 nm (left, middle) and 1 μm (right) (*bottom panel*).
